# Explainable deep drug–target representations for binding affinity prediction

**DOI:** 10.1186/s12859-022-04767-y

**Published:** 2022-06-17

**Authors:** Nelson R. C. Monteiro, Carlos J. V. Simões, Henrique V. Ávila, Maryam Abbasi, José L. Oliveira, Joel P. Arrais

**Affiliations:** 1 Univ Coimbra, Centre for Informatics and Systems of the University of Coimbra, Department of Informatics Engineering, Coimbra, Portugal; 2 BSIM Therapeutics, Instituto Pedro Nunes, Coimbra, Portugal; 3 IEETA, Department of Electronics, Telecommunications and Informatics, University of Aveiro, Aveiro, Portugal

**Keywords:** Drug–target interaction, Binding affinity, Explainable deep learning, Convolutional neural network

## Abstract

**Background:**

Several computational advances have been achieved in the drug discovery field, promoting the identification of novel drug–target interactions and new leads. However, most of these methodologies have been overlooking the importance of providing explanations to the decision-making process of deep learning architectures. In this research study, we explore the reliability of convolutional neural networks (CNNs) at identifying relevant regions for binding, specifically binding sites and motifs, and the significance of the deep representations extracted by providing explanations to the model’s decisions based on the identification of the input regions that contributed the most to the prediction. We make use of an end-to-end deep learning architecture to predict binding affinity, where CNNs are exploited in their capacity to automatically identify and extract discriminating deep representations from 1D sequential and structural data.

**Results:**

The results demonstrate the effectiveness of the deep representations extracted from CNNs in the prediction of drug–target interactions. CNNs were found to identify and extract features from regions relevant for the interaction, where the weight associated with these spots was in the range of those with the highest positive influence given by the CNNs in the prediction. The end-to-end deep learning model achieved the highest performance both in the prediction of the binding affinity and on the ability to correctly distinguish the interaction strength rank order when compared to baseline approaches.

**Conclusions:**

This research study validates the potential applicability of an end-to-end deep learning architecture in the context of drug discovery beyond the confined space of proteins and ligands with determined 3D structure. Furthermore, it shows the reliability of the deep representations extracted from the CNNs by providing explainability to the decision-making process.

**Supplementary Information:**

The online version contains supplementary material available at 10.1186/s12859-022-04767-y.

## Background

In silico methods have been responsible for major advances in the drug discovery field [1]. However, the accurate identification of drug–target interactions (DTIs) remains a decisive turning point in the discovery of new findings and in the understanding of the binding process. Thus, DTI prediction is vital for the development of new drugs, optimizing the whole process chain and leveraging the information available for drug repositioning [2].

Computational DTI prediction methods have been exploiting different properties and an experimentally validated characterization of the interaction to infer new relationships [3]. However, several of these studies rely on binary associations to conduct their experiments, neglecting the importance of the binding affinity. Therefore, the quality of the predictions is usually compromised or at least limited, particularly when considering secondary interactions with off-targets [4]. Furthermore, negative interactions are mostly based on hypotheses, leading to potential false negatives or eventually lack of target selectivity. Despite all these factors, the rise of interactions with known binding affinity measurements has been important to shift computational drug discovery into pursuing the use of these metrics to characterize binding associations, leading to meaningful findings [5]. Nevertheless, binding affinity prediction is substantially more challenging, in which several of these methods have been focusing on the use of biased interaction strengths, encouraging contradictory outcomes.

Deep learning is increasingly being employed in critical contexts such as drug discovery, given its capacity to outperform the traditional machine learning [6]. These architectures have been capable of retrieving unprecedented knowledge in DTIs, and identify complex patterns in drug and protein data collection. However, they are still considered to be opaque and devoided of transparency in their inner operations and results [7, 8]. Thus, it is vital to provide explanations for the reasoning behind the decisions of these architectures, considering that the results presented may have a great impact on the drug discovery pipeline. Nevertheless, the interpretability of these models may present an important opportunity to validate the results and lead to novel findings regarding key regions for the interaction (binding sites) [9].

In this work we make use of an end-to-end deep learning approach to predict drug–target binding affinity measured in terms of the dissociation constant (K_d_), where 1D sequential and structural data, protein sequences and SMILES (Simplified Molecular Input Line Entry System) strings, are used to represent the targets and compounds, respectively. We provide interpretability to the CNNs, present potential explanations for the decisions of the model by showing which inputs regions contributed the most for the predictions, and validate the effectiveness of the deep representations. Overall, in this study we investigate three critical points: a) efficiency of the deep representations in the prediction of a real-valued interaction strength; b) reliability of CNNs in the identification of important sequential and structural regions for binding; and c) robustness of the features extracted from relevant sequential regions.

### Binding affinity prediction

The interaction between an active compound and a protein is determined by comprehensive processes that are heavily reflected on the ligand’s binding affinity and bioactivity [10, 11]. The magnitude and rank order of the binding pair association is usually presented in terms of three different metrics: dissociation constant (K_d_), inhibition constant (K_i_) and half maximal inhibitory concentration (IC_50_) [12]. However, K_d_ is one of the few considered to be unbiased since it is not influenced by the experimental conditions, and expresses a direct measurement of the equilibrium between the receptor-ligand complex and the dissociation components, in which lower values are associated with strong interactions.

Computational drug discovery studies focused in the prediction of binding affinity have been initially driven by the necessity to account for more information in the scoring functions used in structure-based virtual screening. Machine learning methods, including Random Forest, and deep learning architectures, e.g., Feed-Forward Neural Network or 3D CNNs, have been explored as potential replacements for the scoring functions, predicting the binding affinity of protein-ligand complexes either based on different features associated with the 3D structures or 3D single instance learning [13–22]. Apart from improving the scoring functions, some research studies have been pursuing more realistic methodologies to predict DTIs, exploiting the problem as a binding affinity regression task, and making use of chemogenomic and lower structural data, e.g., 1D or 2D data. On that account, three benchmark datasets associated with the studies of Davis et al. [23], Metz et al. [24] and Tang et al. [25], measured in K_d_, K_i_ and KIBA, respectively, have been exploited to conduct the experiments. In addition to some machine learning algorithms, including the Kronecker-regularized least squares [26] or gradient boosting regression trees [27], most of the inferring models have been based on the use of 1D CNNs, 2D CNNs or Graph CNNs, where different representations of the proteins and compounds have been explored, including 1D structures, 2D similarity matrices, feature vectors or even graph representations [28–35].

In spite of the existing binding affinity prediction methodologies, several of these studies still rely on the use of biased binding affinity metrics, i.e., dependent on the measurement conditions, mechanism of inhibition and concentrations [24, 25]. Furthermore, sequential and structural data are still seldomly used together, which is extremely limiting when inferring new interactions, considering that the features used to characterize the DTIs do not reflect the importance of the structural information, specifically related to binding sites. On that account, these models are unable to assess the magnitude of certain local regions in the prediction score. Moreover, binding affinity prediction models based on deep learning have yet to provide explainability to the predictions, compromising the validity of the results, and limiting the identification and comprehension of the underlying aspects around the interaction.

### Explainable deep learning

The interpretability of deep learning architectures have been extremely questioned over the time, especially regarding their intrinsic operations, decisions and results [36]. On that matter, there have been several research advances regarding the explainability of these models and it has been essentially achieved by either adapting the inner architecture (intrinsic interpretability) or performing external evaluations (post-hoc interpretability) [37].

Intrinsic interpretability focus on incorporating explainability directly into the structural units of the architecture (self-interpretable). On that account, attention mechanisms have been explored and incorporated in the architectures to condition the learning process, and provide interpretability through the visualization of the input regions that were given more attention (weight) [38, 39]. Apart from attention layers, local modifications on the units of the architectures, e.g., filters of CNNs, have also been presented to get interpretable knowledge representations [40]. On the other hand, post-hoc interpretability establishes a secondary model in order to provide explanations regarding the model behavior and inner operations. On that matter, methods based on local perturbations, including Prediction Difference Analysis [41] or Occlusion Analysis [42], have been explored to evaluate the model’s response based on general local perturbations, e.g., conditional sampling or masking parts of the input. Furthermore, propagation-based methods have also been considered to be efficient, leveraging the model’s internal structure for the explanation process. On that account, methods such Deconvolution [42], Layer Relevance Propagation [43] or Gradient-Weighted Class Activation Mapping [44], highlight the critical regions in the input for the prediction of the concept, in which the feature activity, the relevance score or the gradients of the model’s outcome are backpropagated to the input domain, respectively.

## Methods

### Binding affinity prediction

#### Drug–target interaction pairs

In order to establish the binding affinity prediction model, we obtained the data from the Davis et al. [23] research study, which comprises selectivity assays related to the human catalytic protein kinome measured in K_d_, resulting in a total of 31,824 interactions between 72 kinase inhibitors (compounds) and 442 kinases (proteins).

The distribution of the binding affinity values is significantly skewed towards K_d_ equal to 10,000 nM (22,400 interaction pairs out of 31,824), which is associated with weak interactions, usually not observed or detected. Furthermore, the variance of this distribution is considerably high, since it ranges from values close to zero (strong interaction) to high values (weak binding). Hence, in order to avoid unnecessary high learning losses, the K_d_ values were transformed into the logarithmic space (pK_d_), spanning from 5 (10,000 nM) to around about 11.

1 $$\begin{aligned} pK_{d} = - log_{10}(\frac{K_{d}}{10^9}) \end{aligned}$$

Davis protein sequences were collected from UniProt [45] using the corresponding accession numbers. Considering that proteins are constituted by an unique amino acid sequence, where each amino acid is considered as a different feature, we have selected only proteins with a length between 264 and 1400 residues (95.7% of the information presented in the dataset) in order avoid increased noise or loss of relevant information. Protein sequences shorter than the maximum length were padded.

The SMILES strings were initially extracted from PubChem [46] based on the compound identifiers. In order to ensure a non data source dependent (consistent) notation to represent the chemical structure of all compounds, RDKit [47] canonical transformation was applied to every SMILES string. Even though the canonical notation does not include stereochemical information, it is a unique representation, where the atoms are consistently numbered. Similar to the protein sequences, we have considered only SMILES with a length between 38 and 72 characters, which corresponds to 95.8% of the information available. SMILES strings shorter than the maximum length were padded.

Table 1 summarizes the number of unique proteins, compounds, and DTIs, as well as the interactions with a pK_d_ value equal to 5 and higher than 5 for the Davis kinase binding affinity dataset before and after pre-processing.

**Table 1:** Davis kinase binding affinity dataset: unique kinases, kinase inhibitors, and DTIs before and after pre-processing

	Proteins	Compounds	DTI	pKd = 5	pKd > 5
*Davis kinase dataset*					
Non-processed	442	72	31,824	22,400	9424
Pre-processed	423	69	29,187	20,479	8708

See Additional file 1: Fig. S1 for more details regarding the Davis dataset.

#### Data representation and encoding

**Fig. 1:**
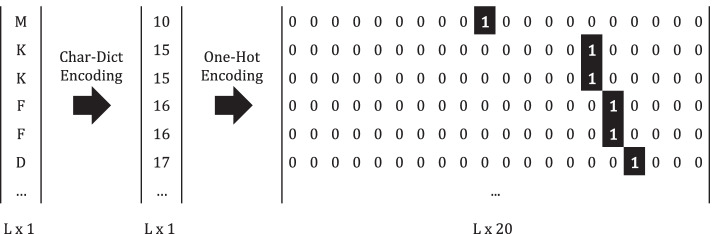
Dictionary-based encoding followed by one-hot encoding applied to the kinase AKK1, where L is the length of the protein sequence

Protein sequences and SMILES strings are constituted by different sequential and structural characters, respectively, which are used as input for the prediction model. A dictionary-based approach was considered to encode each one of the characters into an integer based on the number of different characters, resulting in a 20-character dictionary for the proteins sequences and a 26-character dictionary for the SMILES strings. In order to normalize the importance of each one of these integers values and to preserve only the structural information, a one-hot encoding was applied, assigning a binary variable for each unique integer value, converting every integer into a binary vector. Figure 1 illustrates the dictionary-based approach and the one-hot encoding applied to the AKK1 kinase.

#### Binding affinity prediction model

We make use of an end-to-end deep learning model based on CNNs and Fully-Connected Feed-Forward Neural Networks (FCNNs) to predict a real-valued DTI strength measured in K_d_, where 1D sequential and structural information, protein sequences and SMILES strings, respectively, are used as input.

The architecture of a CNN is organized as a series of layers, comprising convolutional and pooling layers. The convolutional layers are constituted by filters that slide over the input and convolute at each particular location, originating activation maps, which are used as input for the next layer. On the other hand, pooling layers reduce the spatial size of each feature map by replacing local patches of units to a single unit in order to preserve only the features associated with certain motifs rather than its exact location.

**Fig. 2:**
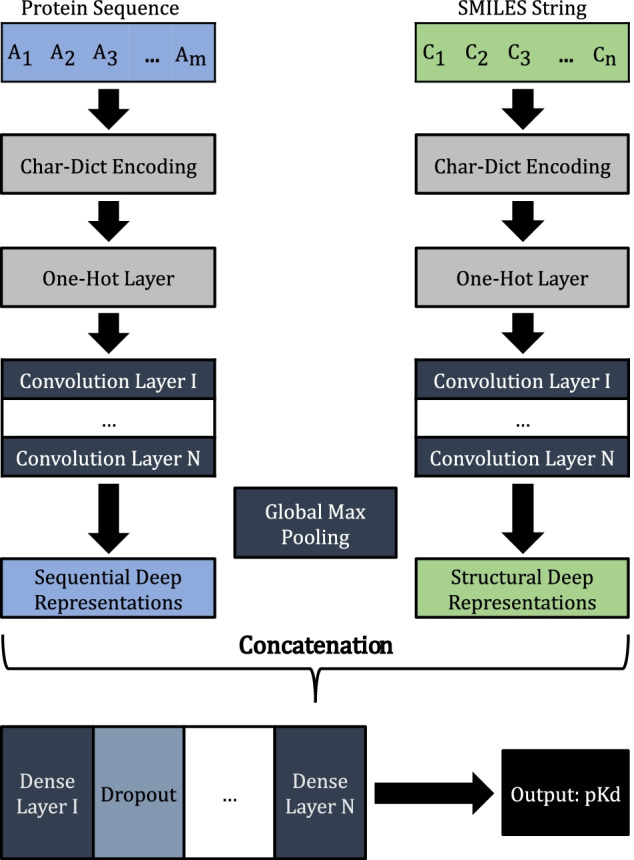
CNN-FCNN binding affinity prediction model

The protein sequences and SMILES strings are initially processed based on their length, and then encoded according to the dictionary-based approach mentioned in the previous section. Considering that these integer values are recognized as categorical variables, an one-hot encoder layer was assigned to both protein sequences and SMILES strings, respectively. Following the one-hot encoding, two parallel series of 1D convolutional layers were considered, one for the protein sequences and the other for the SMILES strings. These series are used to uncover deep patterns in the data, and automatically surmise and identify important sequential and structural regions for the interaction. Global max pooling was also applied, after each series of convolutional layers, in order to reduce the spatial size of each feature map to its maximum representative feature, since we are only interested in the most relevant motifs. The resulting deep representations are then concatenated into a single feature vector, comprising the most relevant sequential and structural features, and used as input for the FCNN, where dropout regularization was applied between each fully-connected layer. The architecture is then followed by an output layer, which is composed by one neuron that returns the real-valued interaction strength measured in pK_d_. The implemented end-to-end deep learning architecture is illustrated in Fig. 2.

#### Chemogenomic representative K-fold

In order to find the best model architecture and set of parameters, we propose a variation of the stratified *K*-fold cross-validation approach. In the context of the problem, a randomly split of the dataset leads to non-representative sets of DTIs, especially when considering an imbalanced distribution of pK_d_ values and that the majority of the interactions are characterized with a pK_d_ value equal to 5 (extremely weak interactions). Moreover, considering that 1D raw data is used to characterize the proteins and compounds, specifically the amino acid sequence and the SMILES string, respectively, the overall representability of the data plays an important role in the learning process of the architecture. Thus, in order to split the dataset into representatives sets, we have considered the proteins similarity, the compounds similarity and also the pK_d_ values distribution during the splitting process.

The proposed method, chemogenomic representative *K*-fold, initially splits the data into two different groups according to the pK_d_ value, specifically higher or equal to 5, respectively. Following the sampling process, the samples with a pK_d_ value superior than 5 are initially distributed across the different *K* folds based on the lowest similarity score (dissimilarity score). The first *K* samples of this group are assigned to each *K* set in order to initialize each fold, and the remaining $N_I - K$ samples ($N_I$ is the number of DTI pairs in the dataset with a pK_d_ value superior than 5) are distributed based on their dissimilarity score. The dissimilarity score corresponds to the lowest similarity score between the sample and each *K* set, in which the sample is assigned to the set with the lowest similarity score. The similarity score is computed as the weighted mean between the median value across all the protein sequences similarity scores and the median value across all the SMILES strings similarity scores, which are calculated (e.g., obtained from similarity matrices) between the sample and each entry in the corresponding set, i.e., between the protein sequence of the sample and all the protein sequences in the corresponding set, and between the SMILES string of the sample and all the SMILES strings in the corresponding set. In order to guarantee that each set is equally sized, only sets that had not previously been assigned a sample are considered at each step (until it is reset), thus, the dissimilarity score corresponds to the lowest similarity convex combination across all $K-m$ sets, where $m=1,..,K-1$ is associated with the number of sets that had previously been assigned a sample. Following the pairs with a pK_d_ value superior than 5, this process is repeated for the remaining $N_{II}$ samples, which correspond to the DTI pairs with a pK_d_ value equal to 5 (weak interactions).

**Fig. 3:**
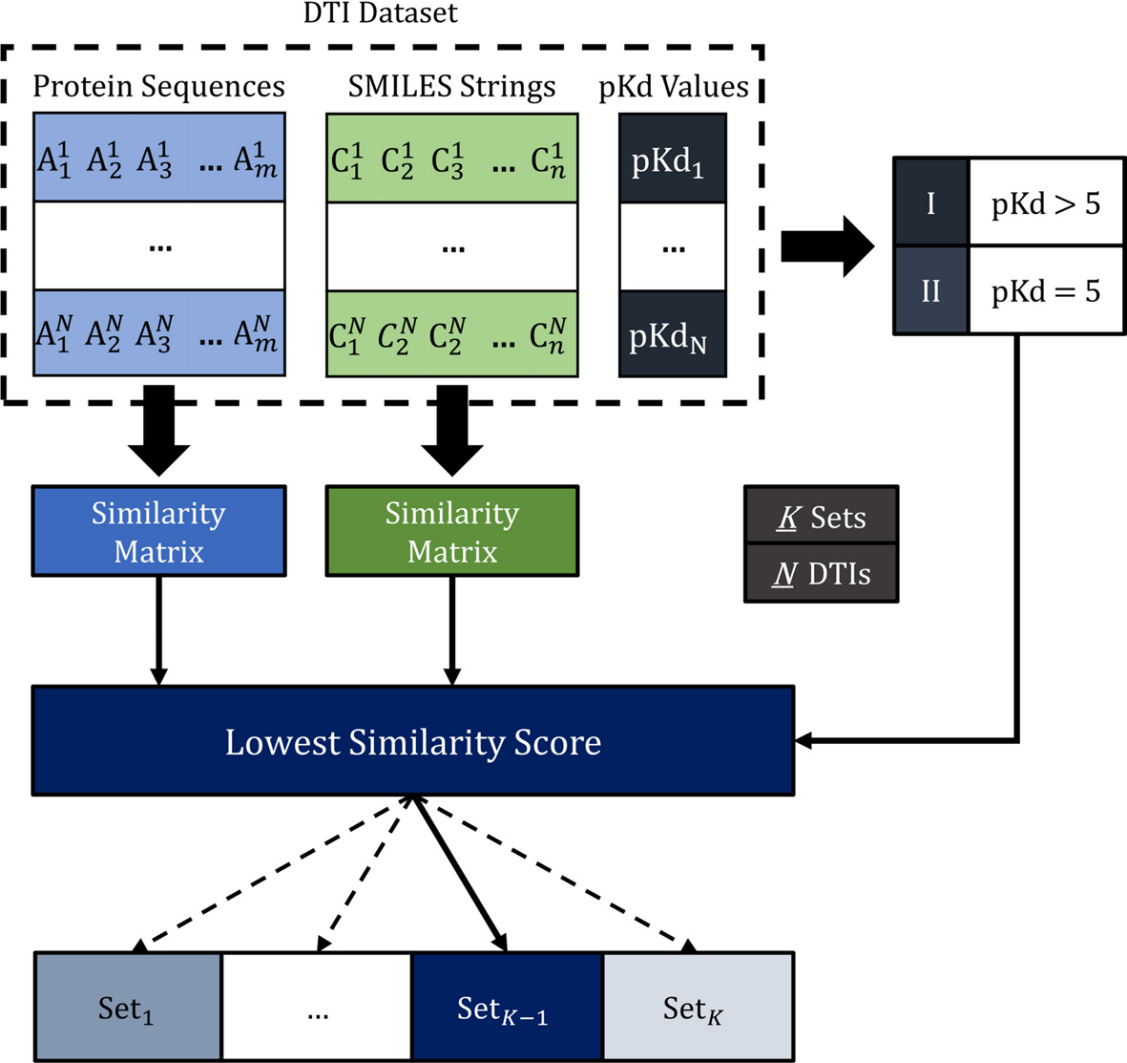
Chemogenomic representative *K*-fold

Overall, this approach leads to equally sized representative sets, prioritizing the relevant interactions. Furthermore, considering that this method splits the data according to the lowest similarity score (improved representability), it is possible to extract an independent testing set in order to evaluate the generalization capacity of the model. The chemogenomic representative *K*-fold is illustrated in Fig. 3.

### Explainable binding affinity prediction

#### Binding sites

The interaction between compounds and proteins results from the recognition and complementarity of certain key groups (binding sites). Considering the range of different regions across the whole structure of proteins and compounds, respectively, the relevance given to certain spots might introduce bias in the predictions. Thus, apart from providing visual explanations to the predictions inferred by the proposed model, it is determinant to evaluate the relevance and significance given to the regions identified as important for the prediction, i.e., the reliability of the model in the identification of binding spots as regions of interest.

We explored the sc-PDB [48] database, which contains some DTI pairs with the interaction regions known, although the number of DTIs with binding sites annotated represents only a small subset of the whole DTI universe. The DTI pairs from this database were initially pre-processed based on the taxonomic identifier (9606 - *Homo sapiens*), protein sequence and SMILES string lengths, and pK_d_ values, where only pairs with a predicted pK_d_ value superior than 5 were considered. The final DTI pairs were then divided into two groups, specifically those that are also present in the Davis dataset (Davis $\cap$ sc-PDB pairs) and the ones that are exclusively from the sc-PDB database. Table 2 summarizes the number of DTIs, the average number of binding sites for each DTI, and the number of unique proteins and compounds for the two datasets.

**Table 2:** Binding sites data collection

	DTI	$\overline{\text{ Binding } \text{ sites }}$	Proteins	Compounds
Davis $\cap$ sc-PDB	32	16	27	8
sc-PDB	266	12	64	249

$\overline{\text{ Binding } \text{ Sites }}$ corresponds to the average number of binding sites annotated for each DTI

#### Position-specific scoring matrices (PSSMs)

Many proteins are functionally and evolutionarily related, where certain regions (motifs/profiles), usually associated with important protein functions/activities, e.g., binding, folding or secondary interactions, are conserved. Hence, in addition to understand if the CNNs are identifying and assigning importance to the binding sites, it is also relevant to explore if there is any association between the input regions selected by the model that are not in the vicinity of the binding regions and the motifs that are usually conserved. In order to obtain the PSSMs, we explored the stand-alone version of PSI-BLAST [49] from blast+ 2.11.0 [50], where the database selected was the non-redundant, the number of iterations was fixed at 3, the E-value chosen was 0.001, and the search was restricted to the taxonomic group 9606. Considering that the PSSM scores range from negative values up to a maximum of 10, we considered different thresholds to select the conserved motifs, specifically from 5 to 10. Table 3 summarizes the average number of conserved motifs for the Davis $\cap$ sc-PDB and sc-PDB pairs across different thresholds.

**Table 3:** Average number of motifs across the different thresholds

	PSSM motifs threshold					
	≥ 5	≥ 6	≥ 7	≥ 8	≥ 9	≥ 10
Davis $\cap$ sc-PDB	276	162	88	45	19	11
sc-PDB	191	121	69	36	15	8

#### Gradient-weighted regression activation mapping

Gradient-Weighted Class Activation Mapping (Grad-CAM) [44] is a gradient-based method that provides visual explanations for the decisions associated with CNN-based architectures, producing coarse localization maps that highlight the important regions for prediction. This method is a generalization of the Class Activation Mapping (CAM) [51] and it uses the gradient information flowing into the last convolutional layer to assign importance to each neuron for a particular decision of interest. The class discriminative localization maps are obtained by performing a linear (weighted) combination of the forward feature maps of the convolutional layer with the neuron importance weights, which is followed by a linear rectification (ReLU) in order to obtain the features that have a positive influence on the class of interest.

2 $$\begin{aligned} L_{Grad-CAM}^{c} \in R^{u x v} = ReLU (\sum _{k}{\alpha _{k}^{c}A^{k}}), \end{aligned}$$

where $L_{Grad-CAM}^{c} \in R^{u x v}$ is the class discriminative localization map of width *u* and height *v* for the class of interest *c*, *k* is the number of feature maps, $A^{k}$ is the *k*th feature map activations, and $\alpha _{k}^{c}$ is the neuron importance weights connecting the *k*th feature map activations with the *c*th class. In order to obtain the neuron importance weights $\alpha _{k}^{c}$, which capture the importance of the feature map *k* for the target class *c*, the gradients of the score for the class of interest ($y_{c}$) with respect to the feature map activations $A^{k}$ of the convolutional layer are computed through backpropagation and global average pooled over the width and height dimensions of the feature map.

3 $$\begin{aligned} \alpha _{k}^{c} = \frac{1}{Z} \sum _{i} \sum _{j} \frac{\partial y^{c}}{\partial A_{ij}^{k}}, \end{aligned}$$

where $\frac{1}{Z} \sum _{i} \sum _{j}$ corresponds to the global average pooling (*Z* is the number of pixels), $\frac{\partial y^{c}}{\partial A_{ij}^{k}}$ to the gradient of the score of class *c* with the respect to the feature map activations $A^{k}$, and *i* and *j* to the width and height dimensions, respectively, of the feature map.

In the context of the problem, we are interested in identifying the discriminative regions towards the regression outcome, specifically the sequential and structural regions in the protein sequences and SMILES strings, respectively, that were considered to be relevant for the prediction of binding affinity. On that account, we present an adaptation of the Grad-CAM approach, where we compute the gradients of the regression outcome with respect to the feature map activations, moving from CAM to Regression Activation Mapping (RAM). Similar to the initial approach, these gradients are global average pooled, resulting in neuron importance weights that capture the importance of the feature map activations for the interaction strength. Overall, this leads to regression discriminative localization maps, capable of explaining the output layer decisions by identifying the relevant sequential and structural regions for prediction.

4 $$\begin{aligned} L_{Grad-RAM} = ReLU (\sum _{k}(\frac{1}{Z} \sum _{i} \sum _{j} \frac{\partial {\hat{y}}}{\partial A_{ij}^{k}})A^{k}), \end{aligned}$$

where $L_{Grad-RAM}$ is the regression discriminative localization map for the predicted value ${\hat{y}}$, $\frac{1}{Z} \sum _{i} \sum _{j}$ corresponds to the global average pooling, $\frac{\partial {\hat{y}}}{\partial A_{ij}^{k}}$ to the gradient of the regression outcome ${\hat{y}}$ with respect to the feature map activations $A^{k}$ of the convolutional layer, and *i* and *j* to the width and height dimensions, respectively, of the feature map.

*Global max pooling* In image detection, global average pooling encourages the network to identify the complete extent of the object, since the average of a feature map takes into account both discriminative and low activation regions. However, in the context of the problem, the interaction is determined by structural and sequential regions scattered in a 1D dimension. Thus, global max pooling is of special interest since our goal is to identify single discriminative spots.

5 $$\begin{aligned} L_{Grad-RAM} = ReLU (\sum _{k}max(\frac{\partial {\hat{y}}}{\partial A^{k}})A^{k}), \end{aligned}$$

where $L_{Grad-RAM}$ is the regression discriminative localization map for the predicted value ${\hat{y}}$, *max* corresponds to the global max pooling, $\frac{\partial {\hat{y}}}{\partial A^{k}}$ to the gradient of the regression outcome ${\hat{y}}$ with respect to the feature map activations $A^{k}$ of the convolutional layer.

*Guided (positive) gradients* In the work of Selvaraju et al. [44], the authors proposed an adaptation of their method by suppressing negative gradients when backpropagating through ReLU layers. Considering that visualizing the sequential and structural regions that have the highest positive influence for the prediction of binding affinity is of special interest, we also explore the results achieved by masking all the gradient positions associated with negative values or where the activations of the feature map are not superior than zero.

6 $$\begin{aligned} \frac{\partial {\hat{y}}}{\partial A^{k}} = (A^{k}> 0) \cdot (\frac{\partial {\hat{y}}}{\partial A^{k}} >0) \cdot \frac{\partial {\hat{y}}}{\partial A^{k}}, \end{aligned}$$

where $\frac{\partial {\hat{y}}}{\partial A^{k}}$ is the gradient of the regression outcome ${\hat{y}}$ with respect to the feature map activations $A^{k}$ of the convolutional layer.

### Experimental setup

#### Binding affinity prediction

The optimized architecture and set of parameters for the proposed model were determined by the chemogenomic *K*-fold cross-validation methodology, which requires a similarity matrix for all the pairs of protein sequences and SMILES strings. Hence, the similarity for the protein pairs was obtained using the Smith-Waterman local alignment algorithm. This method was implemented using the Biostrings R Package [52], where the substitution matrix selected was the BLOSUM62, and the gap penalty for opening and extension was fixed at 10 and 0.5, respectively. Furthermore, the final alignment scores were normalized to a [0,1] range using the approach mentioned in the work of Yamanishi et al. [53]:

7 $$\begin{aligned} SW_{Normalized}(p_1,p_2) = \frac{SW(p_1,p_2)}{\sqrt{SW(p_1,p_1)} * \sqrt{SW(p_2,p_2),}} \end{aligned}$$

where $p_1$ and $p_2$ are the two proteins of a certain pair ($p_1$, $p_2$). On the other hand, the similarity for the SMILES pairs was determined by the Tanimoto Coefficient, where the SMILES strings were initially converted to the Morgan circular fingerprints with a radius of 3, representing the presence or absence of particular substructures across the bitmap. The Tanimoto distance coefficient and the SMILES strings fingerprint transformation were implemented using the RDKit Python package [47]. Consequently, the dataset was splitted into six different folds, in which one fold was used to evaluate the generalization capacity of the model (independent test set) and the remaining folds for hyperoptimization (Additional file 1: Table S1 for more details).

We have hyperoptimized seven parameters: number of convolutional layers, number of dense layers, number of filters for each convolutional layer, filter length, number of neurons for each dense layer, dropout rate and optimizer learning rate. During the cross-validation process, a wide range of values was given for each hyperparameter.

Rectified Linear Unit (ReLU) was selected as the activation function for each convolutional and dense layers, with the exception of the final output layer which uses a linear activation. Additionally, considering that the proposed model focus on a regression task, the loss function selected was the Mean Squared Error (MSE). Regarding the optimizer function, Adaptive Moment Estimation (Adam) was used to update the network weights in each iteration of the learning process.

Furthermore, early stopping with a patience of 30 and model checkpoint were also considered in order to avoid potential overfitting, where the RMSE (Root Mean Squared Error) was evaluated at each epoch by these two callbacks. Overall, the hyperparameter combination that provided the best average RMSE score over the validation sets was selected as the best set of parameters to establish an optimized model and evaluate the generalization capacity on the test set. See Additional file 1: Table S2 for more details regarding the hyperparameters selected.

In order to validate the prediction efficiency of the end-to-end deep learning architecture (CNN-FCNN), we evaluated and compared the performance with different state-of-the-art baselines, specifically KronRLS [26], SimBoost [27], Sim-CNN-DTA [33], DeepDTA [28], DeepCDA [32], and all the different formulations of the GraphDTA [31]. We have followed the same hyperparameter settings described in each one of these papers, with the exception of DeepCDA [32], in which we had to conduct hyperparameter search since the authors did not provide any reference values.

To further evaluate the efficiency of the CNN deep representations, we have compared the performance with Random Forest Regressor (RFR), Support Vector Regressor (SVR), Gradient Boosting Regressor (GBR) and Kernel Ridge Regression (KRR). Scikit-learn [54] was used to implement these models and the parameters were obtained using the chemogenomic *K*-fold cross validation approach (Additional file 1: Table S2 for more details).

We used Python 3.7.9 and Tensorflow 2.4.1 to develop the model, and the experiments were run on 2.20GHz Intel i7-8750H and GeForce GTX 1060 6GB.

#### Explainable binding affinity prediction

We applied Grad-RAM to the implemented trained model, specifically to the last convolutional layers, in order to provide explainability to the predictions by connecting the features extracted from the CNNs to the input domain. However, in the context of the problem, the sole visualization of the input regions that had a positive influence in the prediction does not provide enough significance without any domain knowledge. Thus, we explored the matching and feature relevance correlation between the input regions that had a positive influence in the prediction and the spots associated with binding sites or motifs.

The binding sites (and motifs) are non-consecutive in a 1D representation. Hence, in order to reasonably evaluate the reliability of the CNNs in the identification of these regions as relevant for prediction, we considered the neighborhood of each single position. On that account, for each position *p* associated with a binding (or motif) region, the resulting pocket is given by an interval $]p - s_{w}, p + s_{w}[$, where $s_{w}$ is the size of the window. Nevertheless, the interval is always left or right bounded in the presence of another binding site (or motif) in order to avoid overlapping.

$L_{Grad-RAM}$ *Matching* The regression discriminative localization map provides information regarding the regions of the input that positive-influenced the prediction, and their relative importance (weight). On that account, the first evaluation step consisted in verifying if the CNNs are identifying the binding sites as relevant for the prediction of the binding affinity. We defined different window lengths, ranging from 0 (exact matching) to 5, and evaluated if in these window-based binding pockets the CNNs are extracting information from at least one position, considering that the binding spots are non-consecutive single positions. Furthermore, $L_{Grad-RAM}$ only contains values equal or superior than zero (positive influence). Thus, to evaluate the ${L_{Grad-RAM}}$ matching it is necessary to verify if there is at least one value superior than zero in the window-based binding pocket. Overall, we present this information as matching percentage corresponding to the weighted average of the average number of binding sites, wherein information is being extracted from at least one position, across all the DTI pairs.

8 $$\begin{aligned} \begin{aligned} \sum _{p=1}^{P} \frac{B_{p}}{\sum _{p=1}^{P} B_{p}} * \frac{1}{B_{p}} \sum _{b=1}^{B_{p}} 1, \\ \exists i = 1,...,W : window_{b}(i) > 0, \end{aligned} \end{aligned}$$

where *P* is the number of DTI pairs, *B* is the number of binding sites associated with a certain DTI pair *p*, and *W* is the total length of the window-based pocket.

In the case of the conserved motifs, we also evaluated the $L_{Grad-RAM}$ matching for the positions outside the entire binding region, i.e., from the first to the last binding position.

$L_{Grad-RAM}$*Feature relevance* In addition to the $L_{Grad-RAM}$ matching, it is critical to understand the significance of the features extracted from the window-based pockets, specifically if these features are in the range of those with highest positive influence. On that matter, we defined different thresholds of significance, ranging from the 10% to the 70% highest positive-valued features, in order to perceive what percentage of the features extracted from the window-based pocket regions actually fall into these $L_{Grad-RAM}$ feature threshold distributions. Overall, the $L_{Grad-RAM}$ feature relevance is presented as the weighted average of the average number of positive features extracted from the window-based pocket regions that belong to the feature threshold distribution across all the DTI pairs.

9 $$\begin{aligned} \begin{aligned} \sum _{p=1}^{P} \frac{F_{p}}{\sum _{p=1}^{P} F_{p}} * \frac{1}{F_{P}} \sum _{f=1}^{F_{P}} 1 \iff F_{P}(f) \in \\ \{x \in L_{p}^{GR> 0}||\{y \in L_{p}^{GR> 0}|x \le y\}| \le \lambda |L_{p}^{GR > 0}|\}, \end{aligned} \end{aligned}$$

where *P* is the number of DTI pairs, *F* is the number of positive features extracted from all the window-based pockets, $L_{p}^{GR}$ is the regression discriminative localization map, and $\lambda$ is the significance threshold.

## Results and discussion

### Prediction efficiency of the deep representations

**Table 4:** Binding affinity prediction results of the testing set

Method	Protein Rep.	Compound Rep.	$\downarrow$ MSE	$\downarrow$ RMSE	$\uparrow$ CI	$\uparrow$ $r^{2}$	$\uparrow$ Spearman
*Baseline Methods*							
KronRLS [26]	Smith-Waterman	PubChem-Sim	0.443	0.665	0.847	0.473	0.624
GraphDTA-GCN [31]	1D	Graph	0.315	0.561	0.879	0.625	0.676
GraphDTA-GATNet [31]	1D	Graph	0.307	0.554	0.875	0.634	0.670
SimBoost [27]	Smith-Waterman	PubChem-Sim	0.277	0.526	0.891	0.670	0.694
Sim-CNN-DTA [33]	Smith-Waterman	PubChem-Sim	0.266	0.516	0.884	0.683	0.674
GraphDTA-GIN [31]	1D	Graph	0.255	0.505	0.889	0.696	0.690
GraphDTA-GAT-GCN [31]	1D	Graph	0.254	0.504	0.885	0.697	0.683
DeepDTA [28]	1D	1D	0.222	0.472	0.888	0.735	0.678
DeepCDA [32]	1D	1D	0.202	0.449	0.882	0.760	0.668
*Proposed Method*							
CNN-FCNN	1D	1D	**0.177**	**0.421**	**0.915**	**0.789**	**0.725**
*Deep Representations Eval.*							
SVR	CNN Deep Representations		0.203	0.450	0.907	0.759	0.714
GBR	CNN Deep Representations		0.271	0.520	0.894	0.677	0.699
RFR	CNN Deep Representations		0.283	0.532	0.895	0.663	0.703
KRR	CNN Deep Representations		0.453	0.673	0.848	0.461	0.630

Bold indicates that the best performing values associated with each evaluation metric

RFR, random forest regressor; SVR, support vector regressor; GBR, gradient boosting regressor; KRR, kernel ridge regression

The accurate and reliable prediction of a real-valued interaction strength is a critical point in the path of new findings regarding DTIs. In this study, we make use of an end-to-end deep learning architecture, where CNNs are exploited to automatically identify and extract deep representations from relevant sequential and structural regions. In order to validate the prediction efficiency of the architecture, we evaluated and compared the performance with different state-of-the-art baselines. Additionally, we further validated the efficiency of the features extracted from the CNNs by evaluating and comparing the performance of these deep representations with some baseline models. Table 4 shows the binding affinity prediction results of the testing set in terms of five metrics: MSE, RMSE, Concordance Index (CI), Coefficient of Determination ($r^{2}$) and Spearman Rank Correlation (See Additional file 1: Table S3 for the binding affinity prediction results using the same experimental setup as the state-of-the-art baselines).

The results demonstrate that the CNN-FCNN model achieved the highest performance in terms of MSE (0.177), RMSE (0.421), CI (0.915), Spearman (0.725) and $r^{2}$ (0.789), when compared to state-of-the-art baselines. Hence, it exceeds the other models in its capacity to correctly predict the binding affinity value (lower MSE and RMSE) and distinguish the binding strength rank order across DTI pairs (higher CI).

Regarding the efficiency of the deep representations, the results validate the effectiveness of CNNs in their capacity to extract relevant deep representations from sequential and structural data, especially when considering the performance achieved in terms of CI, which is significantly high across all models and superior than the state-of-the-art baselines (with the exception of the KRR model). Albeit the accurate prediction of the interaction strength value, assessed in terms of MSE and RMSE, is important in the context of the problem, the ability to correctly distinguish the binding strength rank order between two different DTI pairs is of special interest, since it allows to differentiate primary from secondary or not so relevant interactions. On that account, the deep representations extracted from the CNNs are efficient and discriminating in their capacity to describe DTIs and distinguish them based on their binding affinity values.

Additionally, the performance of the SVR model in terms of MSE (0.203), RMSE (0.450), CI (0.907), Spearman (0.714) and $r^{2}$ (0.759) is considerably high and overall superior than all state-of-the-art baselines, despite it being a traditional machine learning approach. These findings demonstrate that the quality and discriminatory power of the input data have a great influence, validating once more the efficiency of the deep representations extracted from the CNNs in the prediction process.

Overall, the use of an end-to-end deep learning architecture to predict binding affinity demonstrates not only the ability of deep learning to automatically identify and extract discriminating features from drug and protein data collection, but also the capacity to learn complex and hidden knowledge related to DTIs for the prediction of binding affinity.

**Fig. 4:**
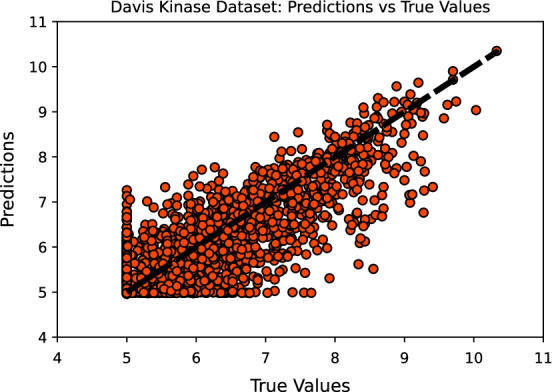
CNN-FCNN model predictions against the true values for the Davis kinase binding affinity testing set, where the diagonal line is the refernece line (*predicted = true value*)

Figure 4 illustrates the predictions from the proposed model against the actual (true) binding affinity values for the Davis testing set, where it is possible to observe a significant density around the *predicted = true value* reference line (perfect model).

### Reliability of the CNNs in the identification of important regions for binding

Despite the prediction efficiency achieved, it is not possible to directly extract explanations for the decision-making process solely based on the deep representations, since they are not (human) interpretable. In this study, we propose Grad-RAM to obtain regression discriminative localization maps, which provide information related to the regions of the input that had a positive influence in the prediction. In order to evaluate the reliability of the CNNs in the identification of important regions for binding, we explored the correlation between the input regions that had a positive influence in the prediction and the window-based pockets related to binding sites and motifs. Table 5 and 6 summarize the $L_{Grad-RAM}$ matching (Eq. 8) for the binding sites of the Davis $\cap$ sc-PDB and sc-PDB pairs, respectively, across different window lengths and for the different formulations of the $L_{Grad-RAM}$.

**Table 5:** Davis $\cap$ sc-PDB Binding Sites - $L_{Grad-RAM}$ matching results (Eq. 8) across different window lengths and for the different formulations of the $L_{Grad-RAM}$, where lower and higher percentage values are associated with lower and higher number of window-based binding pockets where information is being extracted from at least one position across all the DTI pairs, respectively

Window length	GMP-G	GMP-NG	GAP-G	GAP-NG
0	20.74	20.74	20.74	19.57
1	46.32	46.32	46.32	42.83
2	53.29	53.29	53.29	50.39
3	56.98	56.98	56.98	54.07
4	60.66	60.66	60.66	57.95
5	61.24	61.24	61.24	58.72

GMP, global max pooling; GAP, global AVG pooling; G, guided gradients; NG, non guided gradients

**Table 6:** sc-PDB Binding Sites - $L_{Grad-RAM}$ matching results (Eq. 8) across different window lengths and for the different formulations of the $L_{Grad-RAM}$, where lower and higher percentage values are associated with lower and higher number of window-based binding pockets where information is being extracted from at least one position across all the DTI pairs, respectively

Window length	GMP-G	GMP-NG	GAP-G	GAP-NG
0	16.51	16.51	16.51	15.08
1	39.14	39.14	39.14	36.93
2	49.37	49.37	49.37	46.89
3	56.92	56.92	56.92	53.99
4	63.33	63.33	63.33	60.53
5	66.85	66.85	66.85	64.44

GMP, global max pooling; GAP, global AVG pooling; G, guided gradients; NG, non guided gradients

Regarding the differences in the formulation of $L_{Grad-RAM}$, specifically between employing a global max pooling (GMP) instead of a global average pooling (GAP), and between using guided gradients (G) instead of non-guided gradients (NG), the results demonstrate that there is no significant difference, with the exception of the GAP-NG, which generates worse localization maps. On that account, considering that we are interested in the regions with the highest positive influence, we have determined GMP-G to be the most consistent combination, and therefore, used for the evaluations and comparisons.

The Binding sites - $L_{Grad-RAM}$ matching results demonstrate that the CNNs are identifying and extracting features from the window-based binding pockets without any *a priori* information, considering that there is relevant information being detected at every window length. Furthermore, the highest $L_{Grad-RAM}$ matching increase occurs between a window length 0 and 1, and between a window length 1 and 2 (20.74 - 46.32 - 53.29% and 16.51 - 39.14 - 49.37% for the Davis $\cap$ sc-PDB and sc-PDB pairs, respectively), showing that the CNNs are extracting information essentially within the closer regions to the exact binding site location, in which with a window length of 2, the DTI pairs have in average around 50% or more of their window-based binding sites identified. Nevertheless, the $L_{Grad-RAM}$ matching values in the Davis $\cap$ sc-PDB pairs are essentially higher for the lower window lengths when compared to the sc-PDB pairs, which is in agreement with the fact that sc-PDB pairs are not associated only with kinases (representability).

**Fig. 5:**
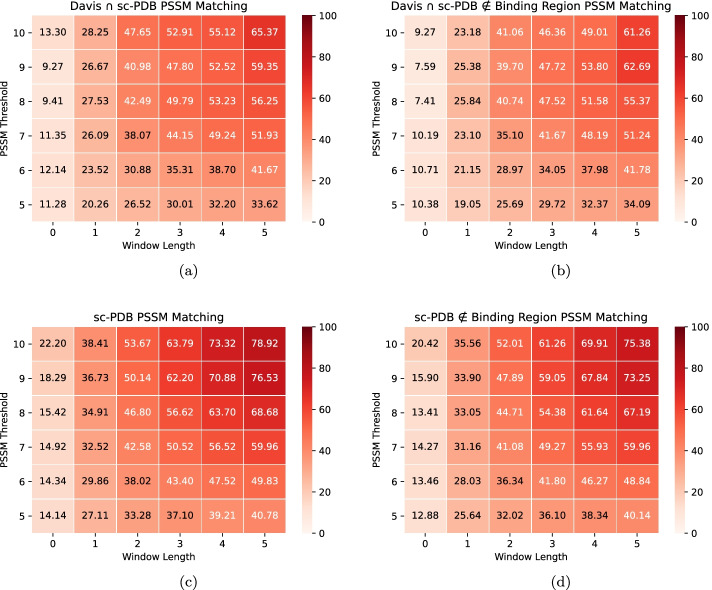
PSSM Motifs - $L_{Grad-RAM}$ matching results (Eq. 8) across different window lengths and PSSM thresholds, where weaker and deeper red colors are associated with lower and higher $L_{Grad-RAM}$ matching values, respectively. **a** Davis $\cap$ sc-PDB pairs; **b** Davis $\cap$ sc-PDB pairs (filtered*); **c** sc-PDB pairs; **d** sc-PDB pairs (filtered*). *Motifs inside the binding region filtered out

Regarding the motifs, the $L_{Grad-RAM}$ matching (Eq. 8) was evaluated across different PSSM thresholds, window lengths, and data collections, where subsets of these datasets, specifically related to the filtering process of the motifs inside the entire binding region, were also considered (Fig. 5). The motifs - $L_{Grad-RAM}$ matching results demonstrate that the CNNs are identifying and extracting features from window-based motifs across different thresholds and window lengths. Similar to the binding sites, the highest $L_{Grad-RAM}$ matching increase occurs between a window length 0 and 1, and between a window length 1 and 2 (e.g., 11.28 - 20.26 - 26.52% for the PSSM threshold 5, and 13.3 - 28.25 - 47.65% for the PSSM threshold 10 for the Davis $\cap$ sc-PDB pairs). The sc-PDB pairs (Fig. 5c, d ) present higher $L_{Grad-RAM}$ matching values, demonstrating that the CNNs are especially focusing on the conserved motifs positions, which reflects the absence of the protein domain similarity. Furthermore, higher PSSM thresholds ($\ge$ 8) are associated with higher $L_{Grad-RAM}$ matching values across the different window lengths, suggesting that the CNNs are focusing on the highly conserved motifs, which are usually associated with important protein functions. Nevertheless, the filtering process of the motifs inside the entire binding region (Fig. 5b, d) resulted in overall lower $L_{Grad-RAM}$ matching values, showing that the CNNs are identifying and extracting features simultaneously from binding sites and motifs.

**Fig. 6:**
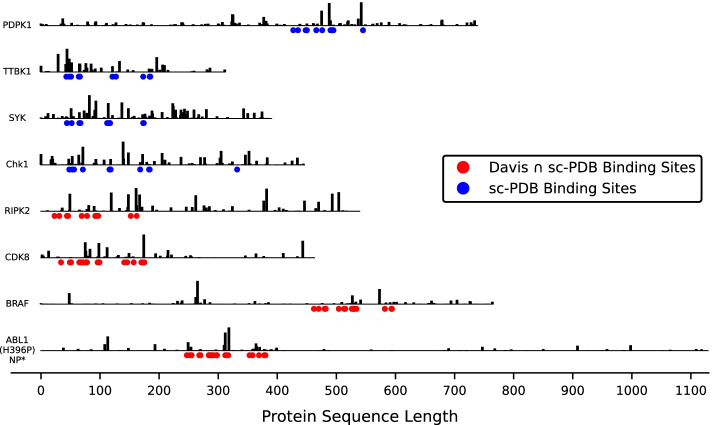
$L_{Grad-RAM}$ maps for some of the protein sequences of the Davis $\cap$ sc-PDB pairs and sc-PDB pairs, where the binding sites are represented by the red and blue circles, respectively. The height of the vertical lines corresponds to importance (weight) of feature extracted from the corresponding position (amino acid).*NP:nonphosphorylated

Figure 6 illustrates the $L_{Grad-RAM}$ maps for some of the protein sequences associated with the Davis $\cap$ sc-PDB and sc-PDB DTI pairs, in which the binding sites are annotated, i.e., known and available.

#### 3D interaction space analysis (docking)

**Fig. 7:**
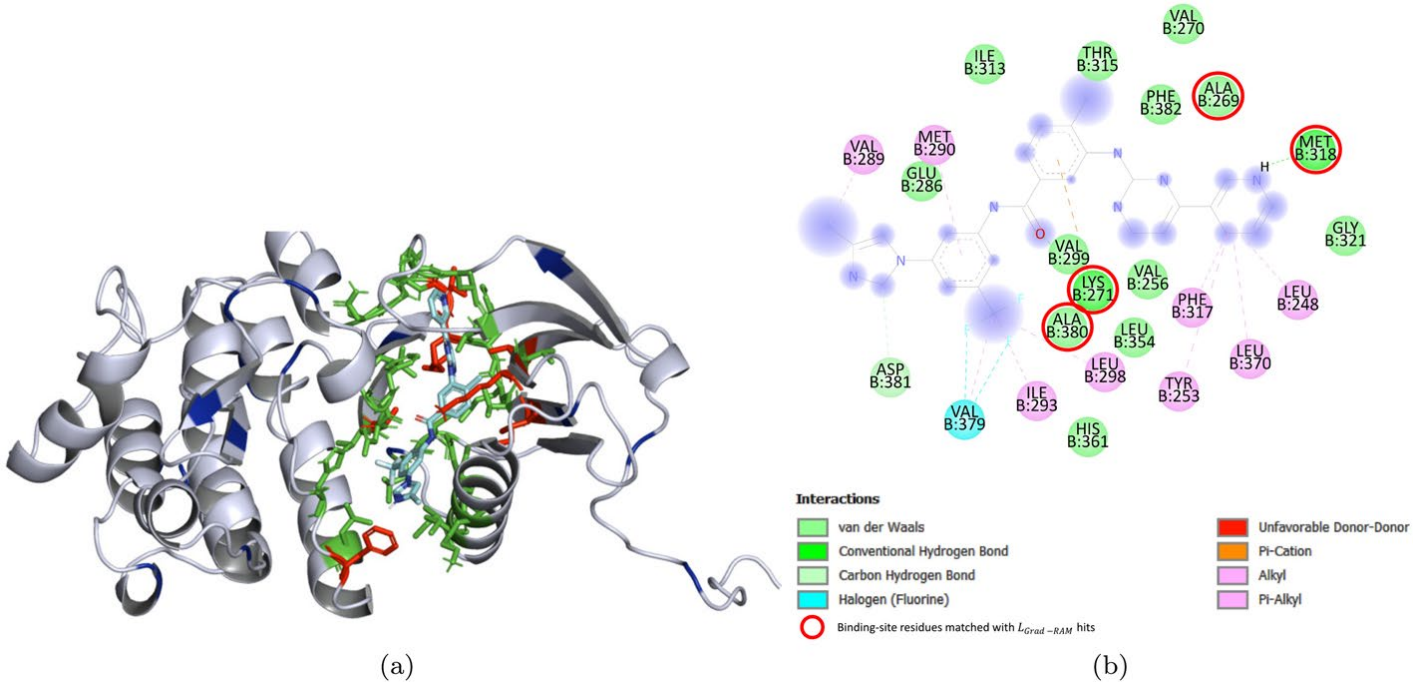
SKI-606 in complex with ABL1(E255K)-phosphorylated. **a** Annotated 3D complex obtained from docking, where the potential binding sites ($\le$ 5 Å), the $L_{Grad-RAM}$ hits, and the matched binding - $L_{Grad-RAM}$ positions are represented by the green, blue and red colors, respectively. **b** 2D Interaction Diagram, in which the matched binding - $L_{Grad-RAM}$ hits are shown delimited by red circles

**Fig. 8:**
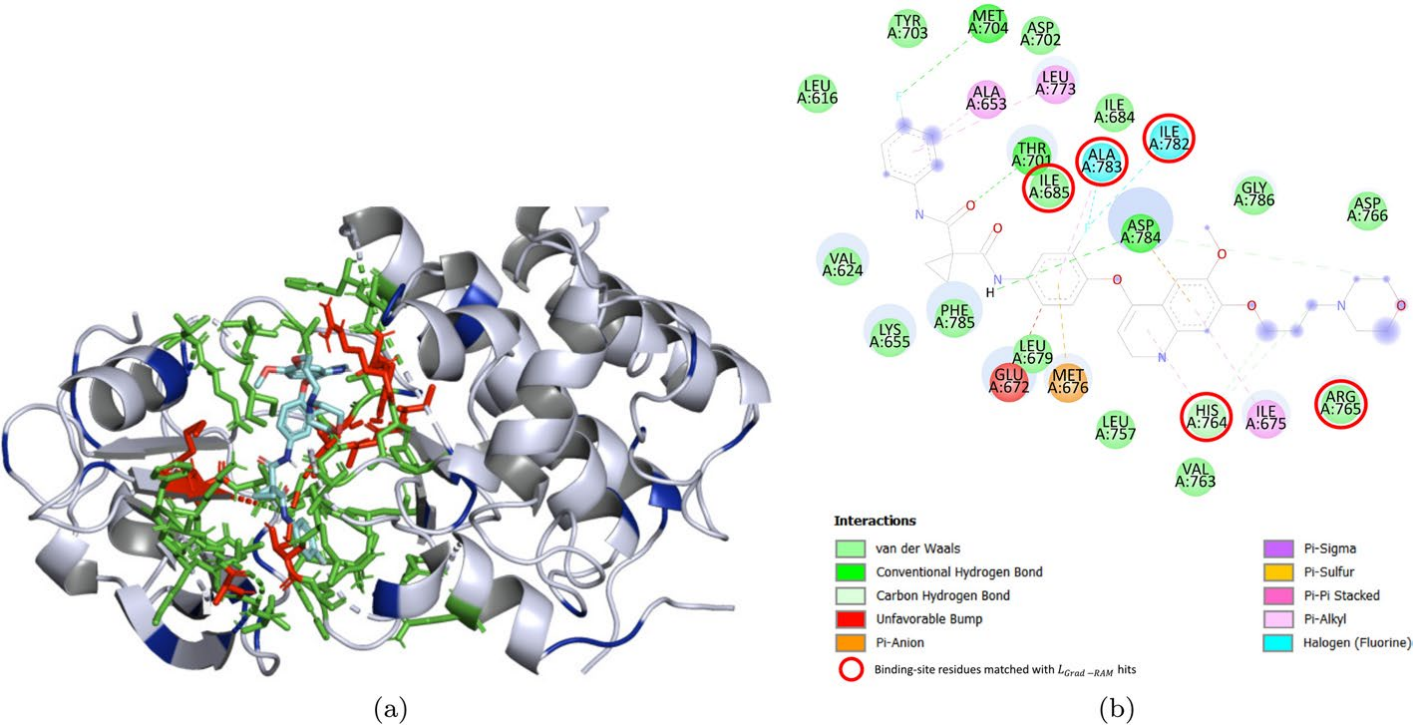
Foretinib in complex with DDR1. **a** Annotated 3D complex obtained from docking, where the potential binding sites ($\le$ 5 Å), the $L_{Grad-RAM}$ hits, and the matched binding - $L_{Grad-RAM}$ positions are represented by the green, blue and red colors, respectively. **b** 2D Interaction Diagram, in which the matched binding - $L_{Grad-RAM}$ hits are shown delimited by red circles

In order to further validate the reliability of the CNNs in the identification of important regions for binding, and the previous Binding sites - $L_{Grad-RAM}$ matching results, we have explored the 3D interaction space for DTI pairs without any binding information available, i.e., where the interacting protein residues are not annotated or available (contrarily to the pairs represented in Fig. 6 and the ones used for the Binding sites - $L_{Grad-RAM}$ matching results). On that account, we have selected two DTI pairs from the Davis kinase binding affinity testing set, specifically ABL1(E255K)-phosphorylated - SKI-606 and DDR1 - Foretinib, and explored the 3D interaction space using docking approaches, wherein the resulting 3D complexes were thoroughly assessed in order to make a fair comparison with the $L_{Grad-RAM}$ hits. Figures 7 and 8 depict the 3D receptor-ligand complex, in which the potential binding sites ($\le$ 5 Å) and the information retrieved from the $L_{Grad-RAM}$ are annotated, and the 2D interaction diagram, where the matched binding - $L_{Grad-RAM}$ positions are annotated, for the ABL1(E255K)-phosphorylated receptor and DDR1 receptor, respectively.

Consistent with the previous findings related to the $L_{Grad-RAM}$ matching results, Figs. 7a and 8a show that the CNNs are not aimlessly identifying regions to extract features from when predicting binding affinity, especially considering that there are $L_{Grad-RAM}$ hits matched with the potential binding sites (also represented in Figs. 7b and 8b ) and others hits near the neighborhood of these interaction spots. Regarding the $L_{Grad-RAM}$ hits close to the main binding pocket and also those not in the vicinity of the binding pocket, their spacial positions suggest they bear relation to conserved regions or other potential interaction pockets/subpockets, e.g., some of these hits are near $\alpha$-helices, which are usually important for the structure and function of the protein, and for certain interactions given their polarity. In particular, for the case of the DDR1 kinase, some of these $L_{Grad-RAM}$ hits were found to be matched or nearly matched with certain experimental validated critical interacting residues.

See Additional file 1: Results Section 3.1.1.1 for more details regarding the docking process and analysis of the resulting 3D complexes.

### Robustness of the deep representations

**Fig. 9:**
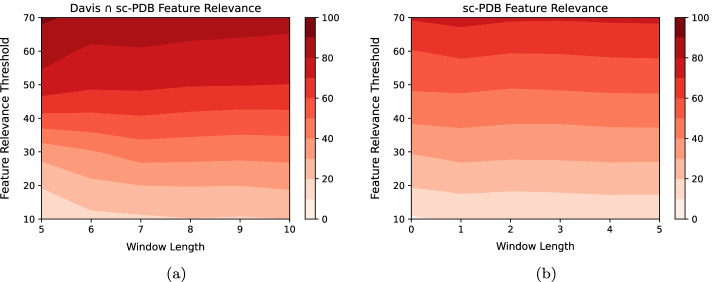
Binding sites - $L_{Grad-RAM}$ feature relevance (Eq. 9) results across different feature relevance thresholds and window lengths, where weaker and deeper red colors are associated with lower and higher $L_{Grad-RAM}$ feature relevance values, respectively. **a** Davis $\cap$ sc-PDB pairs; **b** sc-PDB pairs

In addition to validate the reliability of the CNNs in the identification of important regions for binding, it is critical to understand the robustness (significance) of the deep representations. On that account, we explored the feature relevance correlation between the positive-valued features in the input domain and the ones extracted from the window-based binding sites and motifs, respectively. Figure 9 illustrates the $L_{Grad-RAM}$ Feature Relevance (Eq. 9) in terms of a density map for the binding sites across the different feature relevance thresholds and window length values for the Davis $\cap$ sc-PDB and sc-PDB pairs.

The results demonstrate that at every feature importance threshold and window length value, the Binding sites - $L_{Grad-RAM}$ feature relevance values are superior than the corresponding threshold, i.e., the positive-valued features extracted from the window-based binding pockets are in the range of those with the highest influence. In particular, Fig. 9a shows that at every feature significance threshold, the $L_{Grad-RAM}$ feature relevance value is roughly 10% higher than the corresponding threshold. Regarding the window length, there is no significant difference across the different thresholds, corroborating the Binding sites - $L_{Grad-RAM}$ matching results, where CNNs were shown to extract information within the closer regions to the binding sites. Overall, CNNs are not aimlessly identifying and extracting features from each window-based binding pocket, but essentially assigning significance to these regions when predicting binding affinity.

**Fig. 10:**
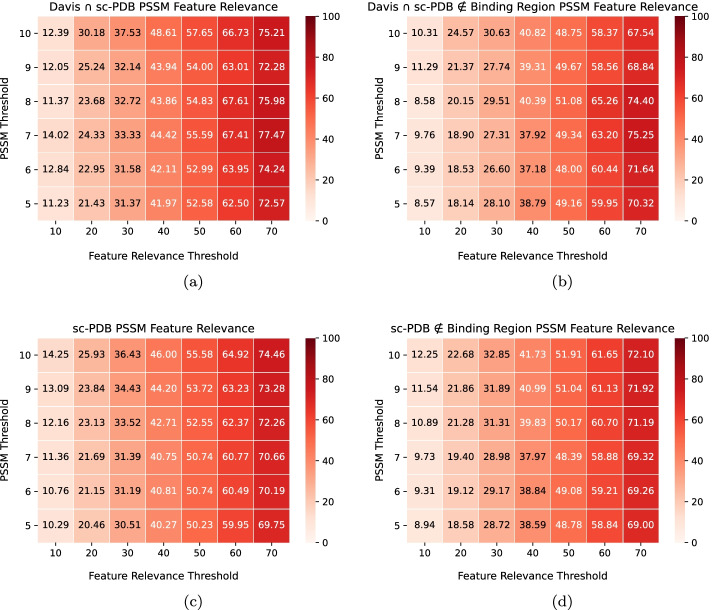
PSSM Motifs - $L_{Grad-RAM}$ feature relevance (Eq. 9) results* across different PSSM thresholds and feature significance thresholds, where weaker and deeper red colors are associated with lower and higher $L_{Grad-RAM}$ matching values, respectively. **a** Davis $\cap$ sc-PDB pairs; **b** Davis $\cap$ sc-PDB pairs (filtered**); **c** sc-PDB pairs; **d** sc-PDB pairs (filtered**). * Each value corresponds to the mean value across the different window lengths. **Motifs inside the binding region filtered out

The motifs - $L_{Grad-RAM}$ feature relevance (Eq. 9) was evaluated across different PSSM thresholds, feature significance thresholds, window lengths and data collections, including the subsets related to the filtering process of the motifs inside the entire binding region. However, since the window length did not represent any significant difference in the results, we considered the mean value across the different window lengths (Fig. 10). The results demonstrate that the CNNs are also assigning significance to the conserved motifs, although inferior than the one given to the window-based binding pockets, considering that the $L_{Grad-RAM}$ feature relevance is essentially lower in filtered pairs and even below the corresponding feature significance threshold values in some cases (illustrated when comparing Fig. 10a–d). Consistent with the previous findings, higher $L_{Grad-RAM}$ feature relevance values are essentially associated with higher PSSM thresholds ($\ge$ 8).

## Conclusion

In this research study, we make use of an end-to-end deep learning architecture to predict drug–target binding affinity measured in K_d_, wherein CNNs are exploited to automatically identify and extract discriminating deep representations from protein sequences and SMILES strings. The deep representations were found to be efficient and discriminating in their capacity to describe DTIs and distinguish them based on their binding affinity values (interaction strength rank order). Furthermore, the CNN-FCNN model yielded better results when compared to state-of-the-art baselines, demonstrating its viability for practical use.

We provide explainability to the predictions by connecting the deep representations extracted from the CNNs to the input domain, exploring the reliability of CNNs in the identification of important sequential regions, specifically binding sites and motifs, when predicting binding affinity. The results demonstrated that the CNNs are identifying and extracting features simultaneously from window-based binding sites and motifs without any *a priori* information. CNNs were found to extract information essentially within the closer regions to the exact binding or motif location, respectively, validating the effectiveness of these architectures in drug discovery. Additionally, we evaluated the significance of the deep representations extracted from these window-based relevant regions for the binding, where the results indicated that the features extracted are in the range of those with the highest positive influence, particularly in the case of the binding sites.

The major contribution of this study relies in an efficient end-to-end deep learning architecture to predict binding affinity beyond the confined space of proteins and ligands with determined 3D structure, in which explanations for the predictions are presented and explored.

Considering the polypharmacology associated with several active small compounds, wherein these drugs interfere with different disease pathways, as future work we will focus on extending this work to validate the identification of important components in the compounds space, which can lead to uncover new off-targets for existing drugs.

## Supplementary Information

**Additional file 1.** Supplement to the main article, containing additional information related to the Methods, and Results and Discussion Sections, respectively. Overall, it comprises the data distributions for the Davis kinase binding affinity dataset, number of DTIs for each chemogenomic representative K-fold resulting fold, the hyperparameters for the proposed model and deep representations baseline models, the details of the binding affinity evaluation metrics, additional details related to the 3D interaction space analysis (docking), and all the L_(Grad-RAM)_ Matching and L_(Grad-RAM)_ Feature Relevance results, respectively.

## Data Availability

The source code and data sets are available at https://github.com/larngroup/Explainable-Deep-DT-Representations.
