# Supplementary material for: Explainable deep drug–target representations for binding affinity prediction
Source: BMC Bioinformatics. 2022 Jun 17;23:237. doi: 10.1186/s12859-022-04767-y (PMC9204982; doi:10.1186/s12859-022-04767-y)
Supplement: Supplementary file 1 — Additional file 1. Supplement to the main article, containing additional information related to the Methods, and Results and Discussion Sections, respectively. Overall, it comprises the data distributions for the Davis kinase binding affinity dataset, number of DTIs for each chemogenomic representative K-fold resulting fold, the hyperparameters for the proposed model and deep representations baseline models, the details of the binding affinity evaluation metrics, additional details related to the 3D interaction space analysis (docking), and all the L(Grad-RAM) Matching and L(Grad-RAM) Feature Relevance results, respectively. [file 12859_2022_4767_MOESM1_ESM.pdf]

Supplement to Explainable Deep Drug-Target Representations for Binding Affinity Prediction

Nelson R. C. Monteiro *[1], Carlos J. V. Simões[2], Henrique V. Ávila[1], Maryam Abbasi[1], José L. Oliveira[3], and Joel P. Arrais[1]

[1]Univ Coimbra, Centre for Informatics and Systems of the University of Coimbra, Department of Informatics Engineering, Coimbra, Portugal
[2]BSIM Therapeutics, Instituto Pedro Nunes, Coimbra, Portugal
[3]IEETA, Department of Electronics, Telecommunications and Informatics, University of Aveiro, Aveiro, Portugal

* E-mail: nelsonrcm@dei.uc.pt

Contents

List of Figures 1

List of Tables 2

1 Supplementary Materials 4
1.1 Davis Kinase Binding Affinity Dataset Distributions 4

2 Supplementary Experimental Setup 5
2.1 Binding Affinity Prediction 5
2.1.1 Binding Affinity Prediction Evaluation Metrics 5

3 Supplementary Results 7
3.1 Binding Affinity Prediction 7
3.2 $L_{Grad-RAM}$ Matching 7
3.2.1 Binding Sites 7
3.2.1.1 3D Interaction Space Analysis (Docking) 7
3.2.2 PSSM Motifs 12
3.2.2.1 Davis - sc-PDB Matched Pairs 12
3.2.2.2 sc-PDB Pairs 13
3.3 $L_{Grad-RAM}$ Feature Relevance 14
3.3.1 Binding Sites 14
3.3.1.1 Davis - sc-PDB Matched Pairs 14
3.3.1.2 sc-PDB Pairs 15
3.3.2 PSSM Motifs 16
3.3.2.1 Davis - sc-PDB Matched Pairs 16
3.3.2.2 sc - PDB Pairs 20

References 24

List of Figures

S1 Davis kinase binding affinity dataset: a) Kd values distribution; b) pKd values distribution; c) pKd $>$ 5 values distribution; d) protein sequences length distribution; e) SMILES string length distribution. 4

S2 Overlapped blind and guided docking best poses: a) SKI-606 (ABL1(E255K) - phosphorylated ligand); b) Foretinib (DDR1 ligand). Blind Docking - Blue, Guided Docking - Orange. 8

S3 Annotated 3D structure for the ABL1(E255K)-phosphorylated receptor in complex with the cognate ligand and docked ligand (SKI-606), where the potential binding sites ($\leq$ 5 Å), the $L_{Grad-RAM}$ hits, the matched binding - $L_{Grad-RAM}$ positions, and the pocket surface are represented by the green, blue, red and orange colors, respectively. a) Full representation of the 3D complex; b) Detail of the pocket surface. Cognate Ligand - Dark Blue, Docked Ligand - Cyan. 9

S4 Annotated 3D structure for the DDR1 receptor in complex with the cognate ligand and docked ligand (Foretinib), where the potential binding sites ($\leq$ 5 Å), the $L_{Grad-RAM}$ hits, the matched binding - $L_{Grad-RAM}$ positions, and the pocket surface are represented by the green, blue, red and orange colors, respectively. a) Full representation of the 3D complex; b) Detail of the pocket surface. Cognate Ligand - Dark Blue, Docked Ligand - Cyan. 9

S5 ABL1(E255K)-phosphorylated 2D Interaction Diagram, in which the binding residues interacting with both the cognate and docked ligands are shown delimited by black circles. a) Cognate Ligand; b) Docked Ligand (SKI-606). 10

S6 DDR1 2D Interaction Diagram, in which the binding residues interacting with both the cognate and docked ligands are shown delimited by black circles. a) Cognate Ligand; b) Docked Ligand (Foretinib). 10

S7 DDR1 kinase domain interactome. a) Interaction map of DDR1.DDR1-pY interactome based on phosphotyrosine peptide pulldowns performed in human placenta tissue [15]; b) Network map of DDR1 interactions, where the interacting residues and the interactors are represented by the yellow and green colors, respectively. 11

List of Tables

S1 Number of DTIs for the different train/validation folds and independent test fold. . . 5

S2 Parameter settings for the proposed model (CNN-FCNN). *Initial number of epochs to allow convergence of the model, where early stopping and model checkpoint were applied to avoid overfitting. 5

S3 Parameters settings for the deep representations evaluation baseline models: a) Random Forest Regressor (RFR); b) Kernel Ridge Regression (KRR); c) Support Vector Regressor (SVR); d) Gradient Boosting Regressor (GBR). 6

S4 The average CI and MSE scores of the test set trained on five different training sets for the Davis dataset. The standard deviations for the proposed method are given in parenthesis. 7

S5 Blind and guided docking scores, measured in terms of kcal/mol, for the best three poses of the ligands associated with each receptor, specifically SKI-606 (ABL1(E255K)-phosphorylated ligand) and Foretinib (DDR1 ligand). 8

S6 PSSM Motifs - $L_{Grad-RAM}$ Matching for the Davis-sc-PDB matched pairs across the different PSSM thresholds: a) PSSM Threshold 5; b) PSSM Threshold 6; c) PSSM Threshold 7; d) PSSM Threshold 8; e) PSSM Threshold 9; f) PSSM Threshold 10. . . 12

S7 PSSM Motifs - $L_{Grad-RAM}$ Matching for the Davis-sc-PDB matched pairs (with the motifs inside the entire binding region filtered out) across the different PSSM thresholds: a) PSSM Threshold 5; b) PSSM Threshold 6; c) PSSM Threshold 7; d) PSSM Threshold 8; e) PSSM Threshold 9; f) PSSM Threshold 10. 12

S8 PSSM Motifs - $L_{Grad-RAM}$ Matching for the sc-PDB pairs across the different PSSM thresholds: a) PSSM Threshold 5; b) PSSM Threshold 6; c) PSSM Threshold 7; d) PSSM Threshold 8; e) PSSM Threshold 9; f) PSSM Threshold 10. 13

S9 PSSM Motifs - $L_{Grad-RAM}$ Matching for the sc-PDB pairs (with the motifs inside the binding region filtered out) across the different PSSM thresholds: a) PSSM Threshold 5; b) PSSM Threshold 6; c) PSSM Threshold 7; d) PSSM Threshold 8; e) PSSM Threshold 9; f) PSSM Threshold 10 13

S10 Binding Sites - $L_{Grad-RAM}$ Feature Relevance for the Davis - sc-PDB pairs across the different feature significance thresholds: a) Feature Relevance 10%; b) Feature Relevance 20%; c) Feature Relevance 30%; d) Feature Relevance 40%; e) Feature Relevance 50%; f) Feature Relevance 60%; g) Feature Relevance 70%. 14

S11 Binding Sites - $L_{Grad-RAM}$ Feature Relevance for the sc-PDB pairs across the different feature significance thresholds: a) Feature Relevance 10%; b) Feature Relevance 20%; c) Feature Relevance 30%; d) Feature Relevance 40%; e) Feature Relevance 50%; f) Feature Relevance 60%; g) Feature Relevance 70%. 15

S12 PSSM Motifs - $L_{Grad-RAM}$ Feature Relevance for the Davis - sc-PDB matched pairs across the different feature significance thresholds, window lengths and PSSM Thresholds: a) PSSM Threshold 5; b) PSSM Threshold 6; c) PSSM Threshold 7. 16

S13 PSSM Motifs - $L_{Grad-RAM}$ Feature Relevance for the Davis - sc-PDB matched pairs across the different feature significance thresholds, window lengths and PSSM Thresholds: a) PSSM Threshold 8; b) PSSM Threshold 9; c) PSSM Threshold 10. 17

S14 PSSM Motifs - $L_{Grad-RAM}$ Feature Relevance for the Davis - sc-PDB matched pairs (with the motifs inside the entire binding region filtered out) across the different feature significance thresholds, window lengths and PSSM Thresholds: a) PSSM Threshold 5; b) PSSM Threshold 6; c) PSSM Threshold 7. 18

S15 PSSM Motifs - $L_{Grad-RAM}$ Feature Relevance for the Davis - sc-PDB matched pairs (with the motifs inside the entire binding region filtered out) across the different feature significance thresholds, window lengths and PSSM Thresholds: a) PSSM Threshold 8; b) PSSM Threshold 9; c) PSSM Threshold 10. 19

S16 PSSM Motifs - $L_{Grad-RAM}$ Feature Relevance for the sc-PDB pairs across the different feature significance thresholds, window lengths and PSSM Thresholds: a) PSSM Threshold 5; b) PSSM Threshold 6; c) PSSM Threshold 7. 20

S17 PSSM Motifs - $L_{Grad-RAM}$ Feature Relevance for the sc-PDB pairs across the different feature significance thresholds, window lengths and PSSM Thresholds: a) PSSM Threshold 8; b) PSSM Threshold 9; c) PSSM Threshold 10. 21
S18 PSSM Motifs - $L_{Grad-RAM}$ Feature Relevance for the sc-PDB pairs (with the motifs inside the entire binding region filtered out) across the different feature significance thresholds, window lengths and PSSM Thresholds: a) PSSM Threshold 5; b) PSSM Threshold 6; c) PSSM Threshold 7. 22
S19 PSSM Motifs - $L_{Grad-RAM}$ Feature Relevance for the sc-PDB pairs (with the motifs inside the entire binding region filtered out) across the different feature significance thresholds, window lengths and PSSM Thresholds: a) PSSM Threshold 8; b) PSSM Threshold 9; c) PSSM Threshold 10. 23

1 Supplementary Materials

1.1 Davis Kinase Binding Affinity Dataset Distributions

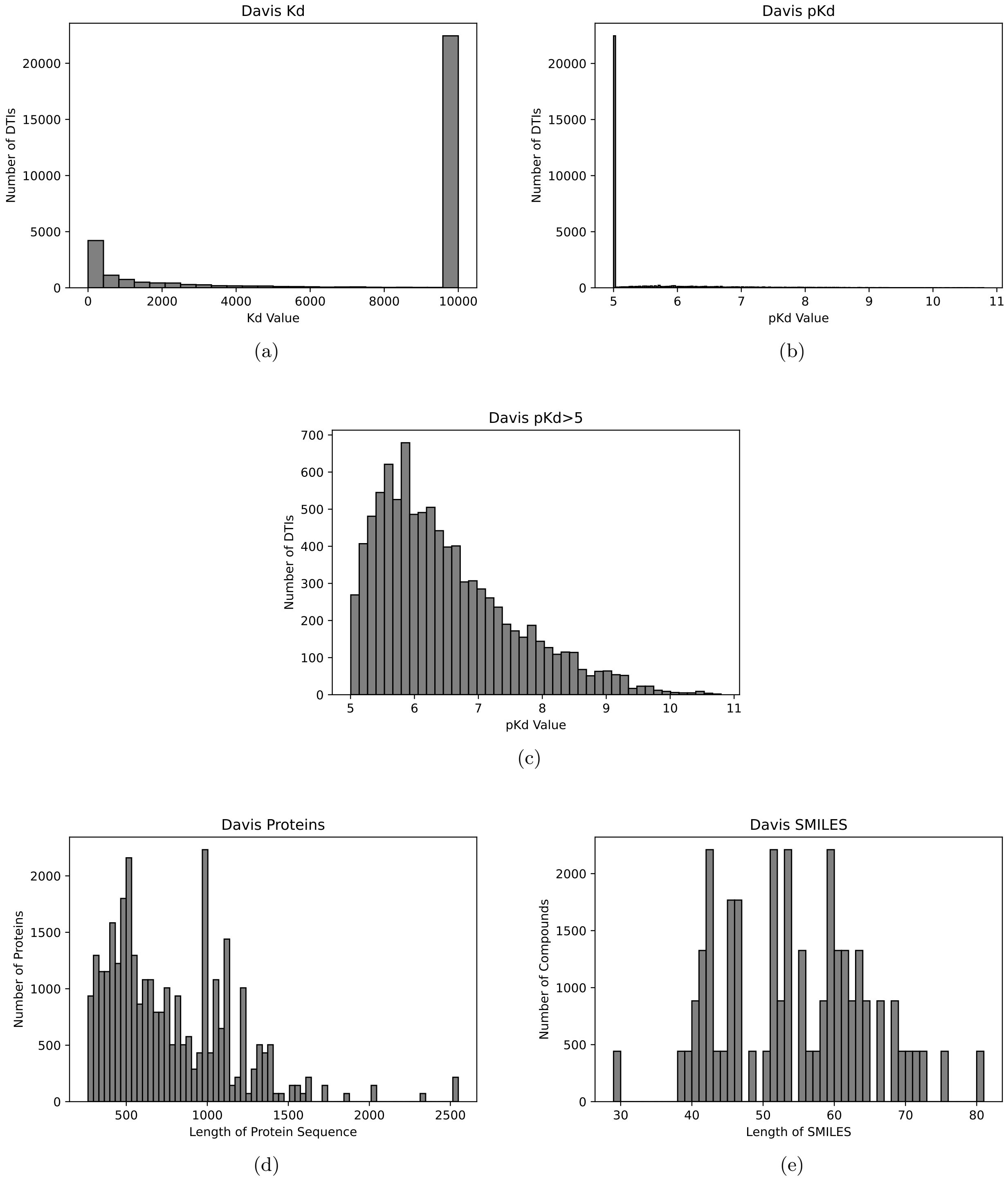

Figure S1: Davis kinase binding affinity dataset: a) Kd values distribution; b) pKd values distribution; c) pKd > 5 values distribution; d) protein sequences length distribution; e) SMILES string length distribution.

2 Supplementary Experimental Setup

2.1 Binding Affinity Prediction

Table S1: Number of DTIs for the different train/validation folds and independent test fold.

	**DTI**	**pKd = 5**	**pKd > 5**
**Train/Validation Fold 0**	4864	3413	1451
**Train/Validation Fold 1**	4864	3412	1452
**Train/Validation Fold 2**	4864	3413	1451
**Train/Validation Fold 3**	4864	3413	1451
**Train/Validation Fold 4**	4864	3413	1451
**Independent Test Fold**	4867	3415	1452

Table S2: Parameter settings for the proposed model (CNN-FCNN). *Initial number of epochs to allow convergence of the model, where early stopping and model checkpoint were applied to avoid overfitting.

**Parameter**	**Value**
**Number of Convolution Layers**	3
**Number of Dense Layers**	3
**Number of Filters**	[64,64,128]
**Filter Length (Proteins)**	[4,4,5]
**Filter Length (Compounds)**	[4,4,5]
**Filter Padding**	'same'
**Hidden Neurons**	[1024,512,1024]
**Dropout Rate**	[0.5,0.1]
**Optimizer**	Adam
**Learning Rate**	1e-04
**Activation Function (CNN)**	ReLU
**Activation Function (FCNN)**	ReLU
**Activation Function (Output)**	Linear
**Loss Function**	MSE
**Epochs***	500

2.1.1 Binding Affinity Prediction Evaluation Metrics

- **Mean Squared Error (MSE)**: measures the average squared difference between the predicted values and the real values.

$$MSE = \frac{1}{n}\sum_{i=1}^{n}(y_i - \hat{y}_i)^2 \tag{1}$$

, where n is the number of samples, y_i the real value and $\hat{y}_i$ the predicted value.

- **Root Mean Squared Error (RMSE)**: measures the square root of the average squared difference between the predicted values and the real values.

$$RMSE = \sqrt{\frac{1}{n}\sum_{i=1}^{n}(y_i - \hat{y}_i)^2} \tag{2}$$

, where n is the number of samples, y_i the real value, and $\hat{y}_i$ the predicted value.

Table S3: Parameters settings for the deep representations evaluation baseline models: a) Random Forest Regressor (RFR); b) Kernel Ridge Regression (KRR); c) Support Vector Regressor (SVR); d) Gradient Boosting Regressor (GBR).

(a)

Parameters	Value
**n_estimators**	300
**criterion**	mse
**max_features**	auto

(b)

Parameters	Value
**alpha**	0.01
**kernel**	poly
**degree**	5

(c)

Parameters	Value
**C**	5
**kernel**	rbf
**gamma**	scale

(d)

Parameters	Value
**n_estimators**	900
**criterion**	friedman_mse
**learning_rate**	0.1
**max_features**	None

- **Concordance Index (CI)**: measures the probability of non-equal pairs being correctly predicted in terms of order.

$$CI = \frac{1}{Z} \sum_{y_i > y_j} h(p_i - p_j), h(p) = \begin{cases} 1, p > 0 \\ 0.5, p = 0 \\ 0, p < 0 \end{cases} \tag{3}$$

, where Z corresponds to the number of non-equal pairs, p_i to the predicted value for the larger affinity y_i, and p_j to the predicted value for the smaller affinity y_j.

- **Coefficient of Determination** (r^2): measures the ratio between the the total variance explained by the model and the total variance.

$$r^2 = 1 - \frac{\sum_{i=1}^{n}(y_i - \hat{y_i})^2}{\sum_{i=1}^{n}(y_i - \overline{y})^2} \tag{4}$$

, where $\hat{y_i}$ is the predicted value, y_i the real value and $\overline{y}$ the mean of the real values.

- **Spearman Rank Correlation** (ρ): measures the strength and direction of association between two ranked variables (non-parametric).

$$Spearman(\rho) = \frac{\frac{1}{n}\sum_{i=1}^{n}(R(y_i) - \overline{R(y)}) \cdot (R(\hat{y_i}) - \overline{R(\hat{y})})}{\sqrt{(\frac{1}{n}\sum_{i=1}^{n}(R(y_i) - \overline{R(y)})^2) \cdot (\frac{1}{n}\sum_{i=1}^{n}(R(\hat{y_i}) - \overline{R(\hat{y})})^2)}} \tag{5}$$

, where $R(\hat{y_i})$ is the predicted value rank, $R(y_i)$ the real value rank, $\overline{R(\hat{y})}$ the mean of the predicted values ranks and $\overline{R(y)}$ the mean of the real values ranks.

3 Supplementary Results

3.1 Binding Affinity Prediction

In order to further the validate the binding affinity performance of the proposed architecture and increase the fairness in the comparisons with the state-of-the-art baseline models, we have also compared the results using the experimental settings (split method) of these baselines. We used the hyperparameters of Table 4 of the main article, and truncated the protein sequences and SMILES strings to the maximum lengths defined in the Subsection "Drug-Target Interaction Pairs" of the main article. Tables S4 reports the average MSE and CI scores over the independent test set using the five different training sets for the Davis dataset.

Table S4: The average CI and MSE scores of the test set trained on five different training sets for the Davis dataset. The standard deviations for the proposed method are given in parenthesis.

Method	Protein Rep.	Compound Rep.	↓ MSE	↑ CI
**Baseline Methods**				
KronRLS [1]	Smith-Waterman	PubChem-Sim	0.379	0.871
Sim-CNN-DTA [2]	Smith-Waterman	PubChem-Sim	0.306	0.855
SimBoost [3]	Smith-Waterman	PubChem-Sim	0.282	0.872
DeepDTA [4]	1D	1D	0.261	0.878
GraphDTA-GCN [5]	1D	Graph	0.254	0.880
DeepCDA [6]	1D	1D	0.248	0.891
GraphDTA-GAT-GCN [5]	1D	Graph	0.245	0.881
GraphDTA-GATNet [5]	1D	Graph	0.232	0.892
GraphDTA-GIN [5]	1D	Graph	0.229	0.893
**Proposed Method**				
**CNN-FCNN**	1D	1D	**0.198 (0.003)**	**0.902 (0.002)**

3.2 $L_{Grad-RAM}$ Matching

3.2.1 Binding Sites

3.2.1.1 3D Interaction Space Analysis (Docking)

Apart from visualization and exploring the $L_{Grad-RAM}$ Matching Results in the 1D space and for DTI pairs with binding information known and available, we have decided to further validate the reliability of the CNNs in the identification of important regions for binding and the Binding sites - $L_{Grad-RAM}$ matching results. On that account, we explored the 3D interaction space for DTI pairs without any interaction information, in which we have selected DTI pairs with a extremely low absolute prediction error from the testing set, specifically ABL1(E255K)-phosphorylated - SKI-606 and DDR1 - Foretinib.

The 3D structures of the proteins were collected from PDB [7], wherein we have selected the 3QRI (`10.2210/pdb3QRI/pdb`) structure for ABL1(E255K)-phosphorylated and the 4BKJ (`10.2210/pdb4BKJ/pdb`) structure for DDR1. These structures were processed using the Discovery Studio Visualizer 4.5 [8] and converted into the PDBQT format using the AutoDockTools 1.5.6 (`https://ccsb.scripps.edu/mgltools/`). On the other hand, OpenBabel 3.1.1 [9] was used to generate the 3D coordinates and convert the SMILES strings into the PDB format. Similar to the proteins, AutoDockTools 1.5.6 (`https://ccsb.scripps.edu/mgltools/`) was used to convert into the PDBQT format.

In order to predict and obtain the resulting receptor-ligand 3D complexes for the two aforementioned DTI pairs, we performed docking experiments using AutoDock Vina 1.2.0 [10]. We have divided the docking process into two different steps: blind docking and guided docking. Regarding the blind docking, we used a search box bigger than the receptors, specifically 60x60x45 Å for ABL1(E255K)-phosphorylated and 75x55x50 Å for DDR1, and an exhaustiveness set to 2000. On the other hand, for the guided docking, we used the DoGSiteScorer [11] platform to obtain an unbiased assessment

of the most likely binding regions (high drug scores), and centered the docking search box around the highest scoring binding pocket (box size of 30x30x30 Å and an exhaustiveness set to 200). The two docking approaches presented very similar results, wherein the RMSD (Root Mean Squared Deviation) between the resulting blind and guided docking best poses is under 0.1 Å for the SKI-606 (ABL1(E255K)-phosphorylated ligand) and equal to 0.2 Å for the Foretinib (DDR1 ligand). Figure S2 illustrates the superimposed blind and guided docking best poses for the ligands associated with each receptor, where it is possible to observe that the best binding poses obtained from each docking approach almost completely overlap.

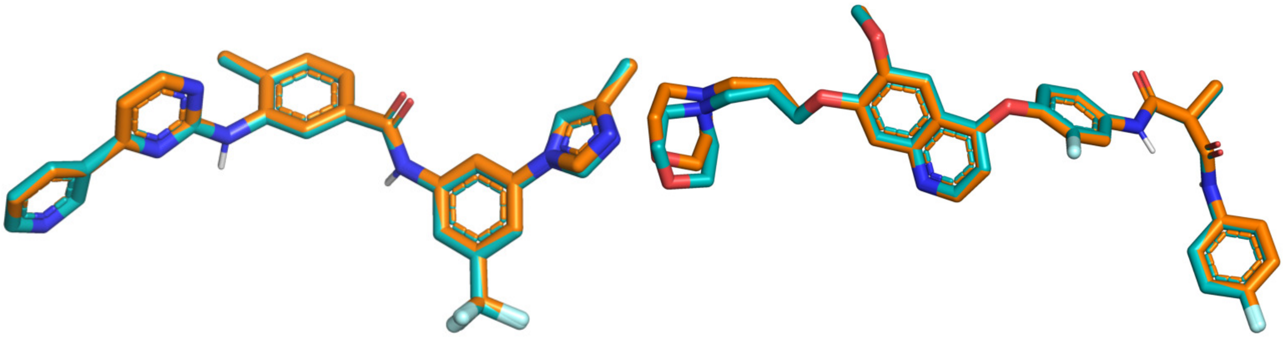

(a) (b)

Figure S2: Overlapped blind and guided docking best poses: a) SKI-606 (ABL1(E255K) - phosphorylated ligand); b) Foretinib (DDR1 ligand). Blind Docking - Blue, Guided Docking - Orange.

Table S5 contains the blind and guided docking scores measured in terms of kcal/mol (binding affinity) for the best three poses of the ligands associated with each receptor, specifically SKI-606 (ABL1(E255K)-phosphorylated ligand) and Foretinib (DDR1 ligand). Consistent with the previous visual findings (Figure S2), the docking score for the best pose of the blind and guided docking methods is in the same strength order for each one of the interaction pairs.

Table S5: Blind and guided docking scores, measured in terms of kcal/mol, for the best three poses of the ligands associated with each receptor, specifically SKI-606 (ABL1(E255K)-phosphorylated ligand) and Foretinib (DDR1 ligand).

		**Binding Affinity (kcal/mol)**	
**Drug-Target Interaction Pair**	**Ligand Pose**	**Blind Docking**	**Guided Docking**
	**1**	-12.4	-12.4
**ABL1(E255K)-phosphorylated - SKI-606**	**2**	-11.6	-11.7
	**3**	-11.1	-11.7
	**1**	-10.8	-10.8
**DDR1 - Foretinib**	**2**	-10.7	-10.7
	**3**	-10.6	-10.7

To further assess the resulting 3D complexes for each one of the selected DTI pairs, we have used the information present in the 3D structures of the receptors in the PDB [7], i.e., the X-ray crystallography structures of ligands in complex with these receptors (cognates). On that account, we have initially evaluated if the pocket surface associated with the X-ray crystallography structure of the ligand in complex with each one of the receptors, which can be considered as a region of high probability of binding, contains the docked ligand, i.e., if it falls inside this binding surface. We have used the the DoGSiteScorer [11] platform to extract the pocket surface, and PyMol [12] for the representation and annotation of these structures.

Considering that we are interested in the binding spots, we have used PyMol [12] to select and identify potential interaction residues in the protein sequences based on a distance equal or lower than 5 Å from the docked ligand molecule. Additionally, since the protein crystal structures from the PDB [7] repository are usually associated with certain fragments of the whole protein 1D amino

acid sequence (e.g., protein sequence from the UniProt database [13]), we had to use BLASTP [14] to align the PDB sequences fragments with the protein sequences used to characterize ABL1(E255K)-phosphorylated and DDR1 in the Davis dataset, respectively. The resulting 3D interaction complexes were then annotated based on the potential binding sites (≤ 5 Å), $L_{Grad-RAM}$ Hits, matched binding-$L_{Grad-RAM}$ positions and pocket surface.

Figures S3 and S4 illustrates the 3D receptor-ligand complexes (both cognate and docked ligand), in which the potential binding sites (≤ 5 Å), the information retrieved from the $L_{Grad-RAM}$ and the pocket surface are annotated.

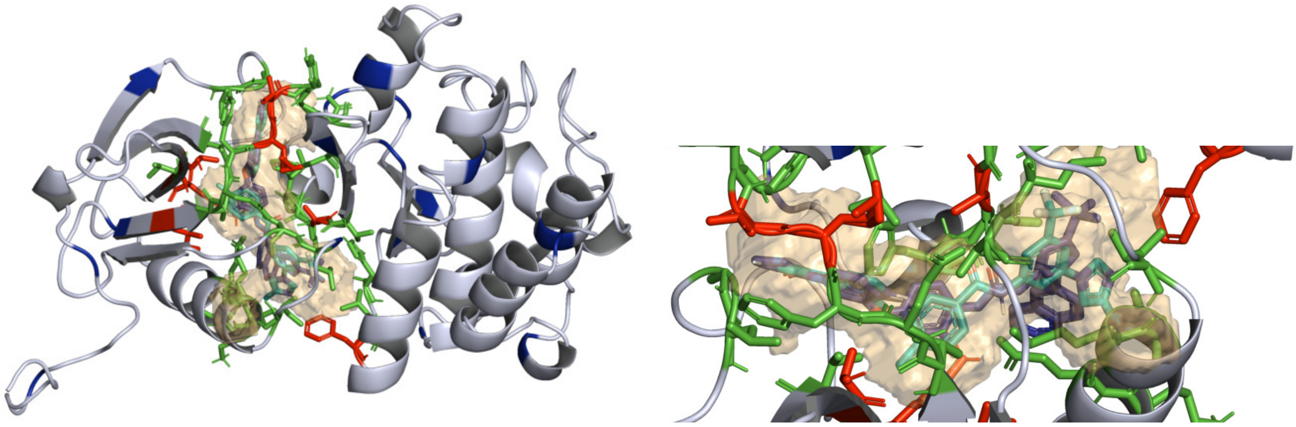

(a) (b)

Figure S3: Annotated 3D structure for the ABL1(E255K)-phosphorylated receptor in complex with the cognate ligand and docked ligand (SKI-606), where the potential binding sites (≤ 5 Å), the $L_{Grad-RAM}$ hits, the matched binding - $L_{Grad-RAM}$ positions, and the pocket surface are represented by the green, blue, red and orange colors, respectively. a) Full representation of the 3D complex; b) Detail of the pocket surface. Cognate Ligand - Dark Blue, Docked Ligand - Cyan.

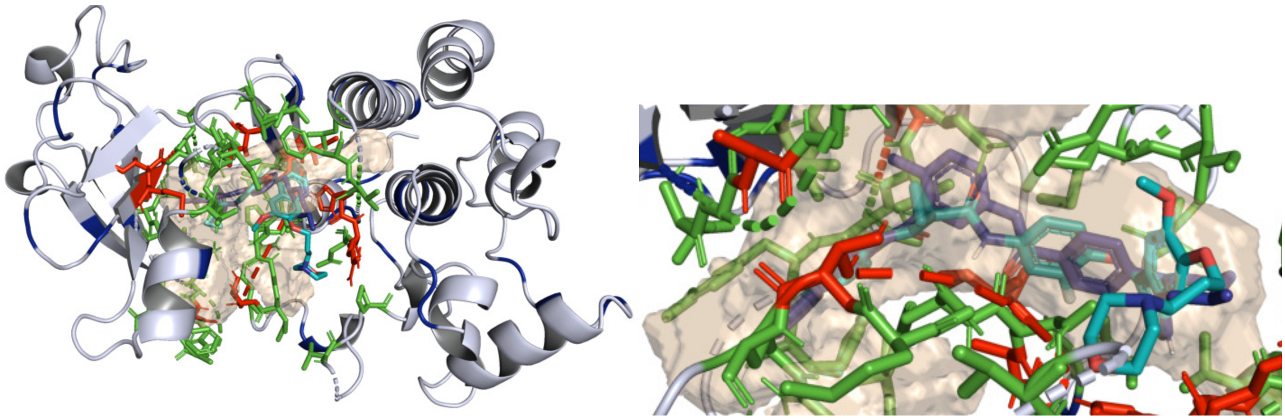

(a) (b)

Figure S4: Annotated 3D structure for the DDR1 receptor in complex with the cognate ligand and docked ligand (Foretinib), where the potential binding sites (≤ 5 Å), the $L_{Grad-RAM}$ hits, the matched binding - $L_{Grad-RAM}$ positions, and the pocket surface are represented by the green, blue, red and orange colors, respectively. a) Full representation of the 3D complex; b) Detail of the pocket surface. Cognate Ligand - Dark Blue, Docked Ligand - Cyan.

These visual findings demonstrate that the docked ligand falls inside the pocket surface associated with the cognate ligand for each one of the receptors considered, which improves the significance of the binding pose of the docked ligands and the overall docking approach. Furthermore, it is also possible to observe in Figures S3b and S4b that the binding poses of the docked ligand and the cognate ligand in each one of the receptors seem to be correlated. On that account, we have decided to explore the correlation of the binding residues associated with each one of these ligands. We used Discovery

Studio Visualizer 4.5 [8] to generate 2D Interaction Diagrams, which represent not only the type of the directed bonds between protein and ligand but also the interacting protein residues. Figures S5 and S6 depicts the 2D Interaction Diagrams for the ABL1(E255K)-phosphorylated receptor in complex with the cognate ligand and the docked ligand, and the DDR1 receptor in complex with the cognate ligand and the docked ligand, respectively.

Figure S5: ABL1(E255K)-phosphorylated 2D Interaction Diagram, in which the binding residues interacting with both the cognate and docked ligands are shown delimited by black circles. a) Cognate Ligand; b) Docked Ligand (SKI-606).

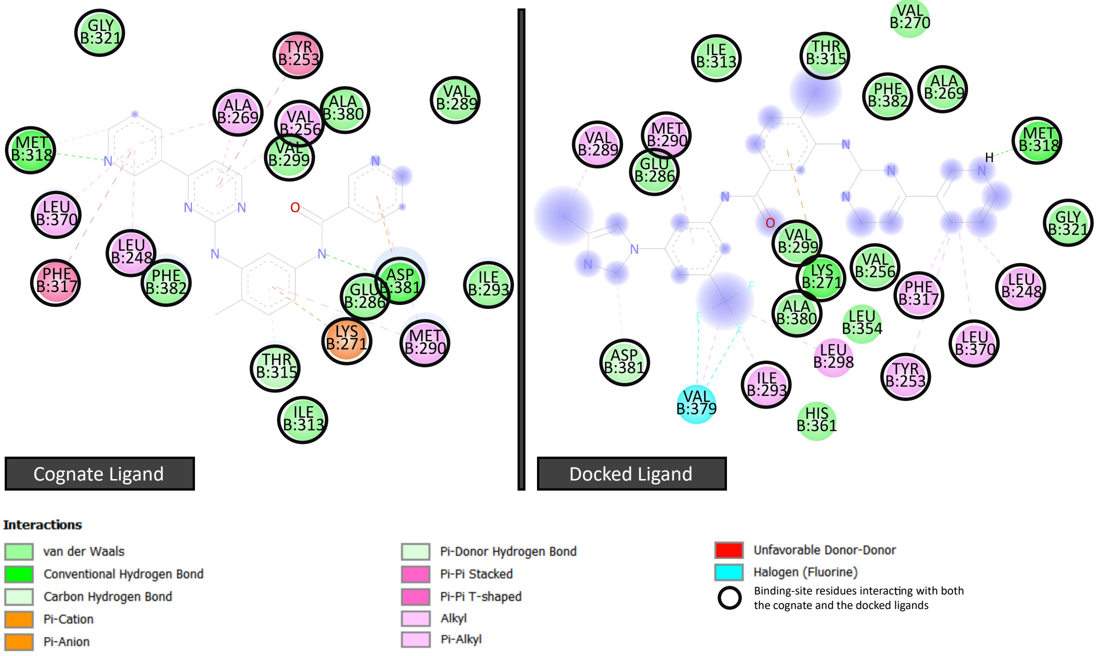

Figure S6: DDR1 2D Interaction Diagram, in which the binding residues interacting with both the cognate and docked ligands are shown delimited by black circles. a) Cognate Ligand; b) Docked Ligand (Foretinib).

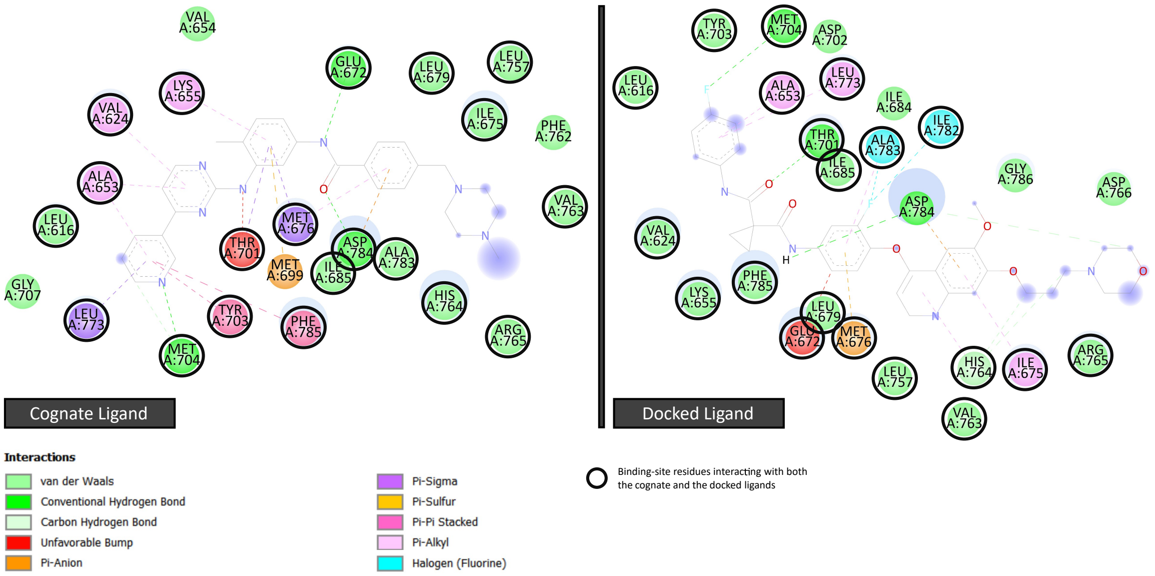

The 2D interaction diagrams for both receptors corroborate the previous visual findings (Figures S3 and S4), where it is possible to observe that the majority of the binding residues are interacting with both the cognate and docked ligands. Overall, these results increase the significance of 3D complexes obtained from docking, and the comparisons with the $L_{Grad-RAM}$ hits.

In addition to exploring the resulting 3D complexes for the two DTI pairs considered, and assessing the visual correlation between the binding sites and $L_{Grad-RAM}$ hits, we have determined to be relevant to check any potential meaning for the $L_{Grad-RAM}$ hits close to the binding pocket and also those not in the vicinity of the binding pocket. In particular, for the DDR1 kinase, which is considered as an important therapeutic target due its implication in pressing contexts, e.g., cancer, we have found that some of these hits are correlated with certain experimental validated critical interacting residues [15], specifically pY703, pY740, pY756, pY792 and pY869. On that account, the $L_{Grad-RAM}$ hits are matched with pY703 (near main binding pocket), pY740 (far away from main binding pocket) and pY869 (far away from main binding pocket), and nearly matched (1 position away) with pY756 and pY792 (far away from binding pocket). Figure S7 illustrates the DDR1 kinase domain interactome related to experimental validated critical interacting residues identified in the research work of Lemeer et al. (2012) [15].

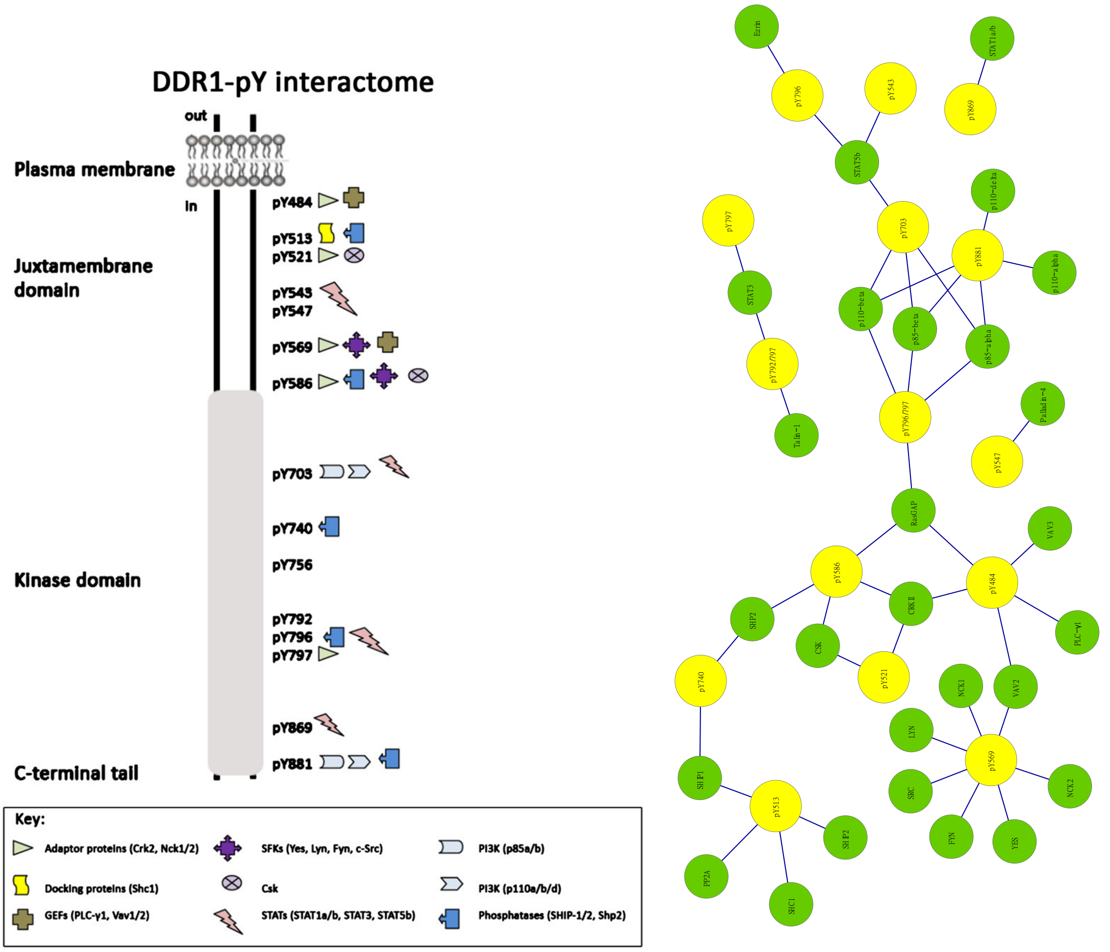

(a) (b)

Figure S7: DDR1 kinase domain interactome. a) Interaction map of DDR1.DDR1-pY interactome based on phosphotyrosine peptide pulldowns performed in human placenta tissue [15]; b) Network map of DDR1 interactions, where the interacting residues and the interactors are represented by the yellow and green colors, respectively.

3.2.2 PSSM Motifs

3.2.2.1 Davis - sc-PDB Matched Pairs

Window Length	GMP-G	GMP-NG	GAP-G	GAP-NG
**0**	11.28	11.28	11.28	10.37
**1**	20.26	20.26	20.26	19.07
**2**	26.52	26.52	26.52	25.11
**3**	30.01	30.01	30.01	28.59
**4**	32.20	32.20	32.20	30.73
**5**	33.62	33.62	33.62	32.12

(a)

Window Length	GMP-G	GMP-NG	GAP-G	GAP-NG
**0**	12.14	12.14	12.14	11.23
**1**	23.52	23.52	23.52	22.40
**2**	30.88	30.88	30.88	29.19
**3**	35.31	35.31	35.31	33.69
**4**	38.70	38.70	38.70	37.24
**5**	41.67	41.67	41.67	40.24

(b)

Window Length	GMP-G	GMP-NG	GAP-G	GAP-NG
**0**	11.35	11.35	11.35	10.39
**1**	26.09	26.09	26.09	25.20
**2**	38.07	38.07	38.07	36.16
**3**	44.15	44.15	44.15	42.42
**4**	49.24	49.24	49.24	47.61
**5**	51.93	51.93	51.93	50.44

(c)

Window Length	GMP-G	GMP-NG	GAP-G	GAP-NG
**0**	9.41	9.41	9.41	7.87
**1**	27.53	27.53	27.53	26.69
**2**	42.49	42.49	42.49	40.17
**3**	49.79	49.79	49.79	48.10
**4**	53.23	53.23	53.23	52.25
**5**	56.25	56.25	56.25	55.41

(d)

Window Length	GMP-G	GMP-NG	GAP-G	GAP-NG
**0**	9.27	9.27	9.27	6.83
**1**	26.67	26.67	26.67	25.37
**2**	40.98	40.98	40.98	37.89
**3**	47.80	47.80	47.80	45.04
**4**	52.52	52.52	52.52	51.06
**5**	59.35	59.35	59.35	58.21

(e)

Window Length	GMP-G	GMP-NG	GAP-G	GAP-NG
**0**	13.30	13.30	13.30	9.42
**1**	28.25	28.25	28.25	26.59
**2**	47.65	47.65	47.65	42.94
**3**	52.91	52.91	52.91	48.48
**4**	55.12	55.12	55.12	53.19
**5**	65.37	65.37	65.37	64.27

(f)

Table S6: PSSM Motifs - $L_{Grad-RAM}$ Matching for the Davis-sc-PDB matched pairs across the different PSSM thresholds: a) PSSM Threshold 5; b) PSSM Threshold 6; c) PSSM Threshold 7; d) PSSM Threshold 8; e) PSSM Threshold 9; f) PSSM Threshold 10.

Window Length	GMP-G	GMP-NG	GAP-G	GAP-NG
**0**	10.38	10.38	10.38	9.50
**1**	19.05	19.05	19.05	17.95
**2**	25.69	25.69	25.69	24.54
**3**	29.72	29.72	29.72	28.51
**4**	32.37	32.37	32.37	31.11
**5**	34.09	34.09	34.09	32.78

(a)

Window Length	GMP-G	GMP-NG	GAP-G	GAP-NG
**0**	10.71	10.71	10.71	9.83
**1**	21.15	21.15	21.15	20.15
**2**	28.97	28.97	28.97	27.69
**3**	34.05	34.05	34.05	32.82
**4**	37.98	37.98	37.98	36.90
**5**	41.78	41.78	41.78	40.74

(b)

Window Length	GMP-G	GMP-NG	GAP-G	GAP-NG
**0**	10.19	10.19	10.19	9.24
**1**	23.10	23.10	23.10	22.10
**2**	35.10	35.10	35.10	33.43
**3**	41.67	41.67	41.67	40.19
**4**	48.19	48.19	48.19	46.86
**5**	51.24	51.24	51.24	50.05

(c)

Window Length	GMP-G	GMP-NG	GAP-G	GAP-NG
**0**	7.41	7.41	7.41	5.78
**1**	25.84	25.84	25.84	25.02
**2**	40.74	40.74	40.74	39.02
**3**	47.52	47.52	47.52	45.80
**4**	51.58	51.58	51.58	50.77
**5**	55.37	55.37	55.37	54.65

(d)

Window Length	GMP-G	GMP-NG	GAP-G	GAP-NG
**0**	7.59	7.59	7.59	4.56
**1**	25.38	25.38	25.38	23.86
**2**	39.70	39.70	39.70	36.23
**3**	47.72	47.72	47.72	44.69
**4**	53.80	53.80	53.80	52.71
**5**	62.69	62.69	62.69	61.61

(e)

Window Length	GMP-G	GMP-NG	GAP-G	GAP-NG
**0**	9.27	9.27	9.27	4.97
**1**	23.18	23.18	23.18	21.52
**2**	41.06	41.06	41.06	36.09
**3**	46.36	46.36	46.36	41.72
**4**	49.01	49.01	49.01	47.35
**5**	61.26	61.26	61.26	59.93

(f)

Table S7: PSSM Motifs - $L_{Grad-RAM}$ Matching for the Davis-sc-PDB matched pairs (with the motifs inside the entire binding region filtered out) across the different PSSM thresholds: a) PSSM Threshold 5; b) PSSM Threshold 6; c) PSSM Threshold 7; d) PSSM Threshold 8; e) PSSM Threshold 9; f) PSSM Threshold 10.

3.2.2.2 sc-PDB Pairs

Window Length	GMP-G	GMP-NG	GAP-G	GAP-NG
**0**	14.14	14.14	14.14	12.84
**1**	27.11	27.11	27.11	24.96
**2**	33.28	33.28	33.28	30.82
**3**	37.10	37.10	37.10	34.54
**4**	39.21	39.21	39.21	36.61
**5**	40.78	40.78	40.78	38.15

(a)

Window Length	GMP-G	GMP-NG	GAP-G	GAP-NG
**0**	14.34	14.34	14.34	13.07
**1**	29.86	29.86	29.86	27.52
**2**	38.02	38.02	38.02	35.32
**3**	43.40	43.40	43.40	40.53
**4**	47.52	47.52	47.52	44.53
**5**	49.83	49.83	49.83	46.82

(b)

Window Length	GMP-G	GMP-NG	GAP-G	GAP-NG
**0**	14.92	14.92	14.92	13.61
**1**	32.52	32.52	32.52	30.12
**2**	42.58	42.58	42.58	39.74
**3**	50.52	50.52	50.52	47.39
**4**	56.52	56.52	56.52	53.29
**5**	59.96	59.96	59.96	56.72

(c)

Window Length	GMP-G	GMP-NG	GAP-G	GAP-NG
**0**	15.42	15.42	15.42	13.97
**1**	34.91	34.91	34.91	32.80
**2**	46.80	46.80	46.80	43.90
**3**	56.62	56.62	56.62	53.22
**4**	63.70	63.70	63.70	60.15
**5**	68.68	68.68	68.68	65.28

(d)

Window Length	GMP-G	GMP-NG	GAP-G	GAP-NG
**0**	18.29	18.29	18.29	16.45
**1**	36.73	36.73	36.73	34.25
**2**	50.14	50.14	50.14	47.36
**3**	62.20	62.20	62.20	58.72
**4**	70.88	70.88	70.88	67.46
**5**	76.53	76.53	76.53	73.36

(e)

Window Length	GMP-G	GMP-NG	GAP-G	GAP-NG
**0**	22.20	22.20	22.20	19.87
**1**	38.41	38.41	38.41	35.23
**2**	53.67	53.67	53.67	50.09
**3**	63.79	63.79	63.79	60.12
**4**	73.32	73.32	73.32	69.87
**5**	78.92	78.92	78.92	75.74

(f)

Table S8: PSSM Motifs - $L_{Grad-RAM}$ Matching for the sc-PDB pairs across the different PSSM thresholds: a) PSSM Threshold 5; b) PSSM Threshold 6; c) PSSM Threshold 7; d) PSSM Threshold 8; e) PSSM Threshold 9; f) PSSM Threshold 10.

Window Length	GMP-G	GMP-NG	GAP-G	GAP-NG
**0**	12.88	12.88	12.88	11.55
**1**	25.64	25.64	25.64	23.57
**2**	32.02	32.02	32.02	29.59
**3**	36.10	36.10	36.10	33.60
**4**	38.34	38.34	38.34	35.80
**5**	40.14	40.14	40.14	37.56

(a)

Window Length	GMP-G	GMP-NG	GAP-G	GAP-NG
**0**	13.46	13.46	13.46	12.12
**1**	28.03	28.03	28.03	25.79
**2**	36.34	36.34	36.34	33.73
**3**	41.80	41.80	41.80	39.06
**4**	46.27	46.27	46.27	43.32
**5**	48.84	48.84	48.84	45.89

(b)

Window Length	GMP-G	GMP-NG	GAP-G	GAP-NG
**0**	14.27	14.27	14.27	12.81
**1**	31.16	31.16	31.16	28.76
**2**	41.08	41.08	41.08	38.30
**3**	49.27	49.27	49.27	46.19
**4**	55.93	55.93	55.93	52.75
**5**	59.96	59.96	59.96	56.72

(c)

Window Length	GMP-G	GMP-NG	GAP-G	GAP-NG
**0**	13.41	13.41	13.41	11.90
**1**	33.05	33.05	33.05	30.94
**2**	44.71	44.71	44.71	41.79
**3**	54.38	54.38	54.38	51.26
**4**	61.64	61.64	61.64	58.29
**5**	67.19	67.19	67.19	63.92

(d)

Window Length	GMP-G	GMP-NG	GAP-G	GAP-NG
**0**	15.90	15.90	15.90	13.99
**1**	33.90	33.90	33.90	31.38
**2**	47.89	47.89	47.89	45.06
**3**	59.05	59.05	59.05	55.79
**4**	67.84	67.84	67.84	64.70
**5**	73.25	73.25	73.25	70.25

(e)

Window Length	GMP-G	GMP-NG	GAP-G	GAP-NG
**0**	20.42	20.42	20.42	17.78
**1**	35.56	35.56	35.56	32.37
**2**	52.01	52.01	52.01	48.41
**3**	61.26	61.26	61.26	57.84
**4**	69.91	69.91	69.91	66.79
**5**	75.38	75.38	75.38	72.31

(f)

Table S9: PSSM Motifs - $L_{Grad-RAM}$ Matching for the sc-PDB pairs (with the motifs inside the binding region filtered out) across the different PSSM thresholds: a) PSSM Threshold 5; b) PSSM Threshold 6; c) PSSM Threshold 7; d) PSSM Threshold 8; e) PSSM Threshold 9; f) PSSM Threshold 10

3.3 $L_{Grad-RAM}$ Feature Relevance

3.3.1 Binding Sites

3.3.1.1 Davis - sc-PDB Matched Pairs

Window Length	GMP-G	GMP-NG	GAP-G	GAP-NG
**0**	13.08	13.08	13.08	12.87
**1**	17.24	17.24	17.24	20.08
**2**	18.42	18.42	18.42	20.62
**3**	19.58	19.58	19.58	21.45
**4**	19.09	19.09	19.09	20.27
**5**	19.84	19.84	19.84	20.83

(a)

Window Length	GMP-G	GMP-NG	GAP-G	GAP-NG
**0**	20.56	20.56	20.56	17.82
**1**	27.59	27.59	27.59	28.03
**2**	30.00	30.00	30.00	30.79
**3**	30.30	30.30	30.30	30.92
**4**	30.08	30.08	30.08	30.96
**5**	31.52	31.52	31.52	32.50

(b)

Window Length	GMP-G	GMP-NG	GAP-G	GAP-NG
**0**	33.64	33.64	33.64	30.69
**1**	39.08	39.08	39.08	39.33
**2**	44.74	44.74	44.74	45.48
**3**	44.06	44.06	44.06	44.89
**4**	43.36	43.36	43.36	44.32
**5**	43.97	43.97	43.97	45.00

(c)

Window Length	GMP-G	GMP-NG	GAP-G	GAP-NG
**0**	57.01	57.01	57.01	58.42
**1**	57.47	57.47	57.47	61.09
**2**	58.95	58.95	58.95	61.86
**3**	57.34	57.34	57.34	60.10
**4**	56.22	56.22	56.22	58.80
**5**	56.61	56.61	56.61	58.96

(d)

Window Length	GMP-G	GMP-NG	GAP-G	GAP-NG
**0**	76.64	76.64	76.64	73.27
**1**	72.03	72.03	72.03	72.80
**2**	72.37	72.37	72.37	72.88
**3**	70.63	70.63	70.63	71.32
**4**	70.33	70.33	70.33	71.05
**5**	69.84	69.84	69.84	70.62

(e)

Window Length	GMP-G	GMP-NG	GAP-G	GAP-NG
**0**	84.11	84.11	84.11	85.15
**1**	78.16	78.16	78.16	79.92
**2**	79.21	79.21	79.21	81.07
**3**	77.62	77.62	77.62	79.55
**4**	76.76	76.76	76.76	78.62
**5**	75.88	75.88	75.88	77.71

(f)

Window Length	GMP-G	GMP-NG	GAP-G	GAP-NG
**0**	91.59	91.59	91.59	93.07
**1**	87.36	87.36	87.36	89.12
**2**	86.84	86.84	86.84	87.57
**3**	85.55	85.55	85.55	86.28
**4**	85.06	85.06	85.06	85.97
**5**	83.85	83.85	83.85	84.58

(g)

Table S10: Binding Sites - $L_{Grad-RAM}$ Feature Relevance for the Davis - sc-PDB pairs across the different feature significance thresholds: a) Feature Relevance 10%; b) Feature Relevance 20%; c) Feature Relevance 30%; d) Feature Relevance 40%; e) Feature Relevance 50%; f) Feature Relevance 60%; g) Feature Relevance 70%.

3.3.1.2 sc-PDB Pairs

**Window Length**	**GMP-G**	**GMP-NG**	**GAP-G**	**GAP-NG**
**0**	8.48	8.48	8.48	8.42
**1**	11.83	11.83	11.83	12.42
**2**	11.85	11.85	11.85	12.65
**3**	11.97	11.97	11.97	12.86
**4**	12.11	12.11	12.11	12.90
**5**	11.82	11.82	11.82	12.54

(a)

**Window Length**	**GMP-G**	**GMP-NG**	**GAP-G**	**GAP-NG**
**0**	20.71	20.71	20.71	20.73
**1**	22.55	22.55	22.55	23.26
**2**	21.69	21.69	21.69	22.47
**3**	22.08	22.08	22.08	22.56
**4**	22.91	22.91	22.91	23.39
**5**	22.81	22.81	22.81	23.11

(b)

**Window Length**	**GMP-G**	**GMP-NG**	**GAP-G**	**GAP-NG**
**0**	30.57	30.57	30.57	30.67
**1**	33.40	33.40	33.40	33.33
**2**	32.45	32.45	32.45	32.59
**3**	32.58	32.58	32.58	32.74
**4**	33.15	33.15	33.15	33.62
**5**	33.03	33.03	33.03	33.35

(c)

**Window Length**	**GMP-G**	**GMP-NG**	**GAP-G**	**GAP-NG**
**0**	41.81	41.81	41.81	43.63
**1**	42.65	42.65	42.65	43.48
**2**	41.53	41.53	41.53	42.46
**3**	41.52	41.52	41.52	42.36
**4**	42.42	42.42	42.42	43.30
**5**	42.57	42.57	42.57	43.16

(d)

**Window Length**	**GMP-G**	**GMP-NG**	**GAP-G**	**GAP-NG**
**0**	51.87	51.87	51.87	52.48
**1**	52.45	52.45	52.45	52.58
**2**	51.05	51.05	51.05	51.60
**3**	51.67	51.67	51.67	52.42
**4**	52.44	52.44	52.44	53.15
**5**	52.62	52.62	52.62	53.20

(e)

**Window Length**	**GMP-G**	**GMP-NG**	**GAP-G**	**GAP-NG**
**0**	59.57	59.57	59.57	59.61
**1**	62.18	62.18	62.18	61.82
**2**	60.56	60.56	60.56	60.74
**3**	60.76	60.76	60.76	61.18
**4**	61.66	61.66	61.66	61.88
**5**	62.00	62.00	62.00	61.98

(f)

**Window Length**	**GMP-G**	**GMP-NG**	**GAP-G**	**GAP-NG**
**0**	71.01	71.01	71.01	71.27
**1**	73.18	73.18	73.18	73.26
**2**	71.27	71.27	71.27	71.90
**3**	70.98	70.98	70.98	71.65
**4**	71.55	71.55	71.55	72.14
**5**	71.79	71.79	71.79	72.14

(g)

Table S11: Binding Sites - $L_{Grad-RAM}$ Feature Relevance for the sc-PDB pairs across the different feature significance thresholds: a) Feature Relevance 10%; b) Feature Relevance 20%; c) Feature Relevance 30%; d) Feature Relevance 40%; e) Feature Relevance 50%; f) Feature Relevance 60%; g) Feature Relevance 70%.

3.3.2 PSSM Motifs

3.3.2.1 Davis - sc-PDB Matched Pairs

	GMP-G							GMP-NG							GAP-G							GAP-NG						
	Feature Relevance Threshold																											
	10	20	30	40	50	60	70	10	20	30	40	50	60	70	10	20	30	40	50	60	70	10	20	30	40	50	60	70
**Window Length** **0**	11.82	22.55	32.06	44.39	56.21	66.53	75.35	11.82	22.55	32.06	44.39	56.21	66.53	75.35	11.82	22.55	32.06	44.39	56.21	66.53	75.35	12.10	22.36	32.50	44.71	56.82	66.85	76.34
**1**	12.17	22.52	32.24	43.10	53.92	63.69	73.30	12.17	22.52	32.24	43.10	53.92	63.69	73.30	12.17	22.52	32.24	43.10	53.92	63.69	73.30	12.47	23.09	33.03	43.65	54.61	64.44	74.04
**2**	11.69	21.94	32.05	43.02	53.50	63.53	74.08	11.69	21.94	32.05	43.02	53.50	63.53	74.08	11.69	21.94	32.05	43.02	53.50	63.53	74.08	11.96	22.47	32.48	43.11	54.22	63.83	74.09
**3**	10.98	21.13	31.40	41.49	51.70	61.75	72.47	10.98	21.13	31.40	41.49	51.70	61.75	72.47	10.98	21.13	31.40	41.49	51.70	61.75	72.47	11.17	21.72	31.72	41.58	52.34	62.20	72.61
**4**	10.53	20.33	30.30	39.95	50.13	59.84	70.17	10.53	20.33	30.30	39.95	50.13	59.84	70.17	10.53	20.33	30.30	39.95	50.13	59.84	70.17	10.40	20.87	30.52	39.99	50.42	60.11	70.20
**5**	10.20	20.10	30.19	39.84	50.01	59.66	70.03	10.20	20.10	30.19	39.84	50.01	59.66	70.03	10.20	20.10	30.19	39.84	50.01	59.66	70.03	10.16	20.64	30.35	39.92	50.28	60.05	70.03

(a)

	GMP-G							GMP-NG							GAP-G							GAP-NG						
	Feature Relevance Threshold																											
	10	20	30	40	50	60	70	10	20	30	40	50	60	70	10	20	30	40	50	60	70	10	20	30	40	50	60	70
**Window Length** **0**	12.38	23.65	31.90	44.60	56.51	68.57	78.73	12.38	23.65	31.90	44.60	56.51	68.57	78.73	12.38	23.65	31.90	44.60	56.51	68.57	78.73	12.52	22.98	32.08	44.25	57.80	69.30	79.25
**1**	13.94	24.21	32.83	43.18	54.20	64.62	73.31	13.94	24.21	32.83	43.18	54.20	64.62	73.31	13.94	24.21	32.83	43.18	54.20	64.62	73.31	14.46	24.04	33.15	43.53	54.87	65.10	73.80
**2**	13.33	23.13	31.16	41.38	52.19	63.49	74.46	13.33	23.13	31.16	41.38	52.19	63.49	74.46	13.33	23.13	31.16	41.38	52.19	63.49	74.46	13.93	23.41	31.79	42.02	53.76	63.87	73.93
**3**	12.59	21.98	31.02	41.11	51.86	62.92	73.80	12.59	21.98	31.02	41.11	51.86	62.92	73.80	12.59	21.98	31.02	41.11	51.86	62.92	73.80	13.06	22.33	31.60	41.44	52.84	63.15	73.08
**4**	12.66	22.55	31.46	41.11	51.66	62.25	73.00	12.66	22.55	31.46	41.11	51.66	62.25	73.00	12.66	22.55	31.46	41.11	51.66	62.25	73.00	12.88	22.78	31.92	41.32	52.39	62.37	72.44
**5**	12.10	22.19	31.12	41.25	51.52	61.82	72.12	12.10	22.19	31.12	41.25	51.52	61.82	72.12	12.10	22.19	31.12	41.25	51.52	61.82	72.12	12.30	22.52	31.50	41.19	52.13	61.75	71.56

(b)

	GMP-G							GMP-NG							GAP-G							GAP-NG						
	Feature Relevance Threshold																											
	10	20	30	40	50	60	70	10	20	30	40	50	60	70	10	20	30	40	50	60	70	10	20	30	40	50	60	70
**Window Length** **0**	6.23	14.95	23.68	36.76	48.29	64.49	76.01	6.23	14.95	23.68	36.76	48.29	64.49	76.01	6.23	14.95	23.68	36.76	48.29	64.49	76.01	8.16	14.29	24.49	38.10	49.66	65.65	78.91
**1**	14.20	25.19	34.57	45.19	56.54	68.52	77.04	14.20	25.19	34.57	45.19	56.54	68.52	77.04	14.20	25.19	34.57	45.19	56.54	68.52	77.04	15.18	25.52	35.21	46.47	57.33	69.11	78.14
**2**	15.38	25.87	35.22	46.46	57.54	68.55	78.58	15.38	25.87	35.22	46.46	57.54	68.55	78.58	15.38	25.87	35.22	46.46	57.54	68.55	78.58	15.95	26.15	35.95	47.53	58.95	69.31	79.11
**3**	16.44	26.46	35.50	46.36	57.16	67.78	77.67	16.44	26.46	35.50	46.36	57.16	67.78	77.67	16.44	26.46	35.50	46.36	57.16	67.78	77.67	16.70	26.60	36.05	46.73	57.73	68.28	77.48
**4**	16.30	27.20	36.11	46.17	57.34	68.03	78.41	16.30	27.20	36.11	46.17	57.34	68.03	78.41	16.30	27.20	36.11	46.17	57.34	68.03	78.41	16.33	27.29	36.63	46.70	58.00	68.34	78.19
**5**	15.58	26.33	34.89	45.56	56.69	67.07	77.13	15.58	26.33	34.89	45.56	56.69	67.07	77.13	15.58	26.33	34.89	45.56	56.69	67.07	77.13	15.66	26.60	35.36	45.60	57.24	67.28	76.78

(c)

Table S12: PSSM Motifs - $L_{Grad-RAM}$ Feature Relevance for the Davis - sc-PDB matched pairs across the different feature significance thresholds, window lengths and PSSM Thresholds: a) PSSM Threshold 5; b) PSSM Threshold 6; c) PSSM Threshold 7.

		GMP-G							GMP-NG							GAP-G							GAP-NG						
		Feature Relevance Threshold																											
		10	20	30	40	50	60	70	10	20	30	40	50	60	70	10	20	30	40	50	60	70	10	20	30	40	50	60	70
Window Length	0	5.97	19.40	26.87	38.06	51.49	66.42	73.88	5.97	19.40	26.87	38.06	51.49	66.42	73.88	5.97	19.40	26.87	38.06	51.49	66.42	73.88	7.14	20.54	31.25	42.86	54.46	70.54	81.25
	1	11.45	23.57	33.04	42.73	52.86	67.40	74.23	11.45	23.57	33.04	42.73	52.86	67.40	74.23	11.45	23.57	33.04	42.73	52.86	67.40	74.23	12.65	24.36	33.49	44.03	53.40	68.38	76.58
	2	11.17	23.51	32.21	43.77	54.81	67.92	76.23	11.17	23.51	32.21	43.77	54.81	67.92	76.23	11.17	23.51	32.21	43.77	54.81	67.92	76.23	11.50	23.28	32.26	44.18	56.24	69.14	78.12
	3	12.80	24.97	34.13	45.68	55.78	67.95	76.90	12.80	24.97	34.13	45.68	55.78	67.95	76.90	12.80	24.97	34.13	45.68	55.78	67.95	76.90	13.01	24.69	34.15	45.49	56.17	68.52	78.42
	4	13.63	25.61	35.50	46.70	57.55	68.92	78.30	13.63	25.61	35.50	46.70	57.55	68.92	78.30	13.63	25.61	35.50	46.70	57.55	68.92	78.30	13.46	25.35	35.67	46.64	57.97	69.31	79.45
	5	13.19	25.00	34.57	46.22	56.48	67.05	76.31	13.19	25.00	34.57	46.22	56.48	67.05	76.31	13.19	25.00	34.57	46.22	56.48	67.05	76.31	13.24	24.84	34.48	45.67	56.78	67.32	77.21

(a)

		GMP-G							GMP-NG							GAP-G							GAP-NG						
		Feature Relevance Threshold																											
		10	20	30	40	50	60	70	10	20	30	40	50	60	70	10	20	30	40	50	60	70	10	20	30	40	50	60	70
Window Length	0	10.53	31.58	36.84	52.63	61.40	66.67	71.93	10.53	31.58	36.84	52.63	61.40	66.67	71.93	10.53	31.58	36.84	52.63	61.40	66.67	71.93	14.29	38.10	47.62	59.52	73.81	83.33	90.48
	1	12.12	27.27	35.35	45.96	55.05	66.16	71.72	12.12	27.27	35.35	45.96	55.05	66.16	71.72	12.12	27.27	35.35	45.96	55.05	66.16	71.72	14.61	29.78	37.64	46.07	57.87	69.66	76.97
	2	10.33	20.67	27.96	40.12	51.06	61.40	71.43	10.33	20.67	27.96	40.12	51.06	61.40	71.43	10.33	20.67	27.96	40.12	51.06	61.40	71.43	11.99	22.26	30.48	40.75	55.14	64.73	76.37
	3	11.22	20.70	27.93	38.15	48.88	58.35	70.07	11.22	20.70	27.93	38.15	48.88	58.35	70.07	11.22	20.70	27.93	38.15	48.88	58.35	70.07	12.78	21.67	29.44	38.33	51.39	60.83	73.33
	4	14.15	25.00	31.98	42.83	53.68	63.18	74.61	14.15	25.00	31.98	42.83	53.68	63.18	74.61	14.15	25.00	31.98	42.83	53.68	63.18	74.61	15.50	25.69	33.76	43.31	55.84	64.76	77.71
	5	13.93	26.23	32.79	43.93	53.93	62.30	73.93	13.93	26.23	32.79	43.93	53.93	62.30	73.93	13.93	26.23	32.79	43.93	53.93	62.30	73.93	14.56	27.00	34.28	44.58	55.77	63.59	76.55

(b)

		GMP-G							GMP-NG							GAP-G							GAP-NG						
		Feature Relevance Threshold																											
		10	20	30	40	50	60	70	10	20	30	40	50	60	70	10	20	30	40	50	60	70	10	20	30	40	50	60	70
Window Length	0	12.50	37.50	43.75	56.25	60.42	66.67	70.83	12.50	37.50	43.75	56.25	60.42	66.67	70.83	12.50	37.50	43.75	56.25	60.42	66.67	70.83	17.65	47.06	58.82	67.65	73.53	82.35	91.18
	1	16.94	41.13	50.00	58.87	65.32	76.61	79.84	16.94	41.13	50.00	58.87	65.32	76.61	79.84	16.94	41.13	50.00	58.87	65.32	76.61	79.84	21.30	46.30	55.56	62.04	70.37	82.41	87.96
	2	10.00	24.55	32.27	43.64	54.55	65.00	75.45	10.00	24.55	32.27	43.64	54.55	65.00	75.45	10.00	24.55	32.27	43.64	54.55	65.00	75.45	12.50	27.08	35.94	46.35	60.42	69.79	83.33
	3	9.96	23.11	30.68	40.64	52.59	62.15	72.51	9.96	23.11	30.68	40.64	52.59	62.15	72.51	9.96	23.11	30.68	40.64	52.59	62.15	72.51	12.22	24.89	33.03	42.53	56.56	66.06	79.64
	4	12.38	26.35	33.33	45.08	55.87	65.08	76.19	12.38	26.35	33.33	45.08	55.87	65.08	76.19	12.38	26.35	33.33	45.08	55.87	65.08	76.19	14.18	28.37	35.82	46.45	59.22	67.73	82.62
	5	12.56	28.46	35.13	47.18	57.18	64.87	76.41	12.56	28.46	35.13	47.18	57.18	64.87	76.41	12.56	28.46	35.13	47.18	57.18	64.87	76.41	13.20	30.34	37.08	48.60	59.55	67.13	81.46

(c)

Table S13: PSSM Motifs - $L_{Grad-RAM}$ Feature Relevance for the Davis - sc-PDB matched pairs across the different feature significance thresholds, window lengths and PSSM Thresholds: a) PSSM Threshold 8; b) PSSM Threshold 9; c) PSSM Threshold 10.

		GMP-G							GMP-NG							GAP-G							GAP-NG						
		Feature Relevance Threshold																											
		10	20	30	40	50	60	70	10	20	30	40	50	60	70	10	20	30	40	50	60	70	10	20	30	40	50	60	70
**Window Length**	**0**	9.97	19.13	28.14	39.89	51.78	62.57	71.31	9.97	19.13	28.14	39.89	51.78	62.57	71.31	9.97	19.13	28.14	39.89	51.78	62.57	71.31	9.85	18.51	28.51	39.85	52.39	62.99	72.39
	**1**	8.32	18.10	28.30	39.20	49.69	60.87	70.86	8.32	18.10	28.30	39.20	49.69	60.87	70.86	8.32	18.10	28.30	39.20	49.69	60.87	70.86	8.36	18.39	28.79	39.34	50.57	61.42	71.51
	**2**	8.64	18.45	28.79	40.07	50.37	61.35	72.33	8.64	18.45	28.79	40.07	50.37	61.35	72.33	8.64	18.45	28.79	40.07	50.37	61.35	72.33	8.74	18.68	29.03	39.54	51.04	61.55	72.22
	**3**	8.40	18.13	28.31	38.72	48.61	59.55	70.77	8.40	18.13	28.31	38.72	48.61	59.55	70.77	8.40	18.13	28.31	38.72	48.61	59.55	70.77	8.43	18.50	28.40	38.34	49.27	60.03	70.87
	**4**	8.15	17.53	27.44	37.36	47.21	57.63	68.30	8.15	17.53	27.44	37.36	47.21	57.63	68.30	8.15	17.53	27.44	37.36	47.21	57.63	68.30	7.80	17.88	27.39	36.89	47.47	57.78	68.21
	**5**	7.93	17.48	27.60	37.48	47.33	57.75	68.37	7.93	17.48	27.60	37.48	47.33	57.75	68.37	7.93	17.48	27.60	37.48	47.33	57.75	68.37	7.70	17.84	27.43	37.10	47.60	57.96	68.17

(a)

		GMP-G							GMP-NG							GAP-G							GAP-NG						
		Feature Relevance Threshold																											
		10	20	30	40	50	60	70	10	20	30	40	50	60	70	10	20	30	40	50	60	70	10	20	30	40	50	60	70
**Window Length**	**0**	10.80	19.25	25.35	37.32	50.00	63.15	74.88	10.80	19.25	25.35	37.32	50.00	63.15	74.88	10.80	19.25	25.35	37.32	50.00	63.15	74.88	10.23	17.39	25.06	35.55	51.41	63.68	74.68
	**1**	8.95	18.67	27.29	37.57	48.29	60.99	70.50	8.95	18.67	27.29	37.57	48.29	60.99	70.50	8.95	18.67	27.29	37.57	48.29	60.99	70.50	9.07	17.79	26.97	36.98	49.35	61.01	70.67
	**2**	9.27	18.31	26.01	36.40	47.09	60.09	72.42	9.27	18.31	26.01	36.40	47.09	60.09	72.42	9.27	18.31	26.01	36.40	47.09	60.09	72.42	9.62	18.03	26.28	36.06	48.80	60.18	71.39
	**3**	8.74	17.25	25.81	36.27	46.96	59.48	71.35	8.74	17.25	25.81	36.27	46.96	59.48	71.35	8.74	17.25	25.81	36.27	46.96	59.48	71.35	8.92	17.27	26.12	35.93	48.01	59.52	70.27
	**4**	9.47	18.84	27.48	37.42	47.62	59.42	70.60	9.47	18.84	27.48	37.42	47.62	59.42	70.60	9.47	18.84	27.48	37.42	47.62	59.42	70.60	9.38	18.82	27.65	37.09	48.45	59.33	69.70
	**5**	9.12	18.87	27.68	38.11	48.05	59.53	70.10	9.12	18.87	27.68	38.11	48.05	59.53	70.10	9.12	18.87	27.68	38.11	48.05	59.53	70.10	9.13	19.03	27.73	37.58	48.79	59.13	69.18

(b)

		GMP-G							GMP-NG							GAP-G							GAP-NG						
		Feature Relevance Threshold																											
		10	20	30	40	50	60	70	10	20	30	40	50	60	70	10	20	30	40	50	60	70	10	20	30	40	50	60	70
**Window Length**	**0**	3.74	8.41	15.89	27.57	41.12	59.35	74.30	3.74	8.41	15.89	27.57	41.12	59.35	74.30	3.74	8.41	15.89	27.57	41.12	59.35	74.30	4.12	6.70	16.49	26.80	42.27	60.31	77.32
	**1**	8.65	18.61	28.01	37.22	49.06	64.10	74.62	8.65	18.61	28.01	37.22	49.06	64.10	74.62	8.65	18.61	28.01	37.22	49.06	64.10	74.62	9.24	18.47	28.31	37.95	50.40	64.86	75.90
	**2**	10.71	20.54	29.02	39.62	50.22	63.28	76.00	10.71	20.54	29.02	39.62	50.22	63.28	76.00	10.71	20.54	29.02	39.62	50.22	63.28	76.00	11.04	20.05	29.29	39.74	51.86	64.23	76.71
	**3**	11.51	20.56	28.91	39.98	50.53	63.01	74.96	11.51	20.56	28.91	39.98	50.53	63.01	74.96	11.51	20.56	28.91	39.98	50.53	63.01	74.96	11.60	20.39	29.28	39.66	51.36	63.80	74.93
	**4**	12.36	23.09	31.61	41.75	52.63	65.06	76.54	12.36	23.09	31.61	41.75	52.63	65.06	76.54	12.36	23.09	31.61	41.75	52.63	65.06	76.54	12.15	22.87	31.86	41.80	53.71	65.46	76.42
	**5**	11.59	22.20	30.40	41.41	52.47	64.39	75.07	11.59	22.20	30.40	41.41	52.47	64.39	75.07	11.59	22.20	30.40	41.41	52.47	64.39	75.07	11.46	22.22	30.57	40.92	53.35	64.60	74.67

(c)

Table S14: PSSM Motifs - $L_{Grad-RAM}$ Feature Relevance for the Davis - sc-PDB matched pairs (with the motifs inside the entire binding region filtered out) across the different feature significance thresholds, window lengths and PSSM Thresholds: a) PSSM Threshold 5; b) PSSM Threshold 6; c) PSSM Threshold 7.

		GMP-G							GMP-NG							GAP-G							GAP-NG						
		Feature Relevance Threshold																											
		10	20	30	40	50	60	70	10	20	30	40	50	60	70	10	20	30	40	50	60	70	10	20	30	40	50	60	70
Window Length	0	4.88	12.20	19.51	28.05	41.46	58.54	68.29	4.88	12.20	19.51	28.05	41.46	58.54	68.29	4.88	12.20	19.51	28.05	41.46	58.54	68.29	6.25	12.50	23.44	29.69	43.75	62.50	76.56
	1	8.58	21.60	31.95	41.72	52.37	68.34	74.85	8.58	21.60	31.95	41.72	52.37	68.34	74.85	8.58	21.60	31.95	41.72	52.37	68.34	74.85	9.75	22.01	32.08	42.14	52.83	69.81	77.36
	2	9.01	22.18	31.37	42.46	52.86	66.72	75.91	9.01	22.18	31.37	42.46	52.86	66.72	75.91	9.01	22.18	31.37	42.46	52.86	66.72	75.91	9.61	21.07	31.05	41.77	53.97	68.39	78.00
	3	9.40	21.23	30.77	42.45	51.99	65.38	75.36	9.40	21.23	30.77	42.45	51.99	65.38	75.36	9.40	21.23	30.77	42.45	51.99	65.38	75.36	9.83	20.27	30.26	41.15	51.89	66.26	77.00
	4	10.35	22.74	32.73	44.40	54.75	67.63	77.38	10.35	22.74	32.73	44.40	54.75	67.63	77.38	10.35	22.74	32.73	44.40	54.75	67.63	77.38	10.55	21.86	32.40	43.58	54.89	68.23	78.91
	5	9.26	20.95	30.74	43.26	53.05	64.95	74.63	9.26	20.95	30.74	43.26	53.05	64.95	74.63	9.26	20.95	30.74	43.26	53.05	64.95	74.63	9.63	20.38	30.23	41.97	53.16	65.45	75.86

(a)

		GMP-G							GMP-NG							GAP-G							GAP-NG						
		Feature Relevance Threshold																											
		10	20	30	40	50	60	70	10	20	30	40	50	60	70	10	20	30	40	50	60	70	10	20	30	40	50	60	70
Window Length	0	5.71	11.43	14.29	31.43	45.71	54.29	60.00	5.71	11.43	14.29	31.43	45.71	54.29	60.00	5.71	11.43	14.29	31.43	45.71	54.29	60.00	9.52	9.52	19.05	38.10	57.14	76.19	90.48
	1	12.41	25.52	33.79	45.52	57.24	66.90	72.41	12.41	25.52	33.79	45.52	57.24	66.90	72.41	12.41	25.52	33.79	45.52	57.24	66.90	72.41	15.63	28.13	36.72	46.88	60.94	72.66	80.47
	2	10.97	20.68	27.43	38.40	47.68	57.38	69.20	10.97	20.68	27.43	38.40	47.68	57.38	69.20	10.97	20.68	27.43	38.40	47.68	57.38	69.20	13.59	22.33	30.58	39.81	51.94	62.14	76.21
	3	12.12	20.88	27.61	36.36	45.12	53.54	67.68	12.12	20.88	27.61	36.36	45.12	53.54	67.68	12.12	20.88	27.61	36.36	45.12	53.54	67.68	14.50	22.14	29.39	37.02	47.71	56.87	72.14
	4	14.06	25.00	32.03	41.93	51.30	60.42	72.40	14.06	25.00	32.03	41.93	51.30	60.42	72.40	14.06	25.00	32.03	41.93	51.30	60.42	72.40	16.09	25.57	33.91	43.10	53.45	62.64	76.44
	5	12.47	24.73	31.29	42.23	50.98	58.86	71.33	12.47	24.73	31.29	42.23	50.98	58.86	71.33	12.47	24.73	31.29	42.23	50.98	58.86	71.33	14.08	25.30	32.70	43.44	52.74	60.86	74.70

(b)

		GMP-G							GMP-NG							GAP-G							GAP-NG						
		Feature Relevance Threshold																											
		10	20	30	40	50	60	70	10	20	30	40	50	60	70	10	20	30	40	50	60	70	10	20	30	40	50	60	70
Window Length	0	7.14	14.29	17.86	32.14	39.29	50.00	53.57	7.14	14.29	17.86	32.14	39.29	50.00	53.57	7.14	14.29	17.86	32.14	39.29	50.00	53.57	13.33	13.33	26.67	40.00	46.67	66.67	86.67
	1	17.24	39.08	47.13	56.32	64.37	73.56	75.86	17.24	39.08	47.13	56.32	64.37	73.56	75.86	17.24	39.08	47.13	56.32	64.37	73.56	75.86	23.29	45.21	54.79	61.64	71.23	82.19	87.67
	2	9.87	23.68	29.61	38.16	46.05	56.58	69.08	9.87	23.68	29.61	38.16	46.05	56.58	69.08	9.87	23.68	29.61	38.16	46.05	56.58	69.08	13.28	26.56	34.38	42.19	52.34	62.50	79.69
	3	9.55	21.91	28.09	35.39	44.38	53.93	66.29	9.55	21.91	28.09	35.39	44.38	53.93	66.29	9.55	21.91	28.09	35.39	44.38	53.93	66.29	12.50	23.68	30.92	38.16	48.68	58.55	75.66
	4	9.72	24.07	30.56	40.74	48.61	58.33	69.91	9.72	24.07	30.56	40.74	48.61	58.33	69.91	9.72	24.07	30.56	40.74	48.61	58.33	69.91	12.17	25.93	33.33	43.39	52.38	61.90	78.31
	5	8.36	24.36	30.55	42.18	49.82	57.82	70.55	8.36	24.36	30.55	42.18	49.82	57.82	70.55	8.36	24.36	30.55	42.18	49.82	57.82	70.55	10.12	25.91	32.39	44.53	52.23	60.73	76.92

(c)

Table S15: PSSM Motifs - $L_{Grad-RAM}$ Feature Relevance for the Davis - sc-PDB matched pairs (with the motifs inside the entire binding region filtered out) across the different feature significance thresholds, window lengths and PSSM Thresholds: a) PSSM Threshold 8; b) PSSM Threshold 9; c) PSSM Threshold 10.

3.3.2.2 sc - PDB Pairs

		GMP-G							GMP-NG							GAP-G							GAP-NG						
		Feature Relevance Threshold																											
		10	20	30	40	50	60	70	10	20	30	40	50	60	70	10	20	30	40	50	60	70	10	20	30	40	50	60	70
Window Length	0	10.72	21.02	31.02	40.75	50.19	59.68	69.61	10.72	21.02	31.02	40.75	50.19	59.68	69.61	10.72	21.02	31.02	40.75	50.19	59.68	69.61	10.76	20.98	30.92	40.79	50.36	59.41	69.36
	1	10.76	20.98	31.23	40.91	50.78	60.52	70.22	10.76	20.98	31.23	40.91	50.78	60.52	70.22	10.76	20.98	31.23	40.91	50.78	60.52	70.22	10.79	21.08	31.28	41.08	51.20	60.66	70.43
	2	10.34	20.40	30.47	40.17	50.08	59.84	69.55	10.34	20.40	30.47	40.17	50.08	59.84	69.55	10.34	20.40	30.47	40.17	50.08	59.84	69.55	10.37	20.44	30.57	40.27	50.27	59.95	69.69
	3	10.13	20.30	30.33	40.18	50.30	60.01	69.78	10.13	20.30	30.33	40.18	50.30	60.01	69.78	10.13	20.30	30.33	40.18	50.30	60.01	69.78	10.18	20.33	30.39	40.21	50.43	60.07	69.86
	4	9.97	20.12	30.10	39.92	50.11	59.88	69.66	9.97	20.12	30.10	39.92	50.11	59.88	69.66	9.97	20.12	30.10	39.92	50.11	59.88	69.66	9.98	20.12	30.12	39.87	50.15	59.89	69.71
	5	9.82	19.94	29.89	39.70	49.92	59.77	69.68	9.82	19.94	29.89	39.70	49.92	59.77	69.68	9.82	19.94	29.89	39.70	49.92	59.77	69.68	9.84	19.99	29.93	39.73	49.98	59.81	69.76

(a)

		GMP-G							GMP-NG							GAP-G							GAP-NG						
		Feature Relevance Threshold																											
		10	20	30	40	50	60	70	10	20	30	40	50	60	70	10	20	30	40	50	60	70	10	20	30	40	50	60	70
Window Length	0	10.46	20.96	30.77	40.63	50.57	60.08	69.98	10.46	20.96	30.77	40.63	50.57	60.08	69.98	10.46	20.96	30.77	40.63	50.57	60.08	69.98	10.60	20.98	30.75	40.97	51.00	60.11	69.84
	1	11.56	22.01	32.24	41.75	51.78	61.38	70.85	11.56	22.01	32.24	41.75	51.78	61.38	70.85	11.56	22.01	32.24	41.75	51.78	61.38	70.85	11.54	22.13	32.33	41.96	52.19	61.53	71.01
	2	11.08	21.40	31.52	41.08	50.91	60.82	70.35	11.08	21.40	31.52	41.08	50.91	60.82	70.35	11.08	21.40	31.52	41.08	50.91	60.82	70.35	11.14	21.60	31.74	41.21	51.22	60.96	70.54
	3	10.73	21.16	31.14	40.75	50.64	60.49	70.24	10.73	21.16	31.14	40.75	50.64	60.49	70.24	10.73	21.16	31.14	40.75	50.64	60.49	70.24	10.80	21.28	31.17	40.74	50.70	60.49	70.15
	4	10.47	20.79	30.80	40.38	50.31	60.10	69.81	10.47	20.79	30.80	40.38	50.31	60.10	69.81	10.47	20.79	30.80	40.38	50.31	60.10	69.81	10.55	20.93	30.80	40.30	50.33	60.06	69.79
	5	10.26	20.61	30.68	40.30	50.24	60.09	69.89	10.26	20.61	30.68	40.30	50.24	60.09	69.89	10.26	20.61	30.68	40.30	50.24	60.09	69.89	10.34	20.67	30.64	40.18	50.24	60.01	69.83

(b)

		GMP-G							GMP-NG							GAP-G							GAP-NG						
		Feature Relevance Threshold																											
		10	20	30	40	50	60	70	10	20	30	40	50	60	70	10	20	30	40	50	60	70	10	20	30	40	50	60	70
Window Length	0	10.95	20.53	29.56	39.07	49.33	59.45	69.97	10.95	20.53	29.56	39.07	49.33	59.45	69.97	10.95	20.53	29.56	39.07	49.33	59.45	69.97	11.33	20.65	30.08	39.87	49.82	59.34	69.60
	1	12.06	22.15	31.62	40.84	50.65	60.89	70.67	12.06	22.15	31.62	40.84	50.65	60.89	70.67	12.06	22.15	31.62	40.84	50.65	60.89	70.67	12.11	22.17	31.62	40.92	50.75	60.58	70.64
	2	11.74	22.47	32.49	41.70	51.48	61.42	71.16	11.74	22.47	32.49	41.70	51.48	61.42	71.16	11.74	22.47	32.49	41.70	51.48	61.42	71.16	11.91	22.53	32.59	41.89	51.54	61.21	71.17
	3	11.58	22.17	32.01	41.37	51.18	61.13	70.90	11.58	22.17	32.01	41.37	51.18	61.13	70.90	11.58	22.17	32.01	41.37	51.18	61.13	70.90	11.71	22.31	32.01	41.55	51.30	61.01	70.75
	4	11.06	21.62	31.53	40.96	51.05	60.96	70.76	11.06	21.62	31.53	40.96	51.05	60.96	70.76	11.06	21.62	31.53	40.96	51.05	60.96	70.76	11.26	21.85	31.55	41.09	51.06	60.77	70.67
	5	10.78	21.20	31.12	40.58	50.73	60.74	70.49	10.78	21.20	31.12	40.58	50.73	60.74	70.49	10.78	21.20	31.12	40.58	50.73	60.74	70.49	10.95	21.33	31.09	40.64	50.81	60.65	70.45

(c)

Table S16: PSSM Motifs - $L_{Grad-RAM}$ Feature Relevance for the sc-PDB pairs across the different feature significance thresholds, window lengths and PSSM Thresholds: a) PSSM Threshold 5; b) PSSM Threshold 6; c) PSSM Threshold 7.

		GMP-G							GMP-NG							GAP-G							GAP-NG						
		Feature Relevance Threshold																											
		10	20	30	40	50	60	70	10	20	30	40	50	60	70	10	20	30	40	50	60	70	10	20	30	40	50	60	70
**Window Length**	**0**	13.30	25.05	36.60	45.67	55.07	64.27	73.94	13.30	25.05	36.60	45.67	55.07	64.27	73.94	13.30	25.05	36.60	45.67	55.07	64.27	73.94	13.49	25.13	37.29	46.63	55.00	64.20	73.09
	**1**	13.06	24.16	34.40	43.43	53.62	63.50	73.17	13.06	24.16	34.40	43.43	53.62	63.50	73.17	13.06	24.16	34.40	43.43	53.62	63.50	73.17	12.95	23.97	34.19	43.39	53.10	62.76	72.28
	**2**	12.31	23.07	33.18	42.27	52.23	62.20	72.30	12.31	23.07	33.18	42.27	52.23	62.20	72.30	12.31	23.07	33.18	42.27	52.23	62.20	72.30	12.17	22.99	33.04	42.39	51.95	61.76	71.59
	**3**	11.83	22.51	32.65	41.88	51.58	61.44	71.47	11.83	22.51	32.65	41.88	51.58	61.44	71.47	11.83	22.51	32.65	41.88	51.58	61.44	71.47	11.74	22.56	32.41	41.96	51.50	61.26	70.87
	**4**	11.34	22.16	32.32	41.67	51.54	61.33	71.28	11.34	22.16	32.32	41.67	51.54	61.33	71.28	11.34	22.16	32.32	41.67	51.54	61.33	71.28	11.44	22.31	32.17	41.71	51.45	61.12	70.89
	**5**	11.16	21.85	31.97	41.36	51.28	61.46	71.42	11.16	21.85	31.97	41.36	51.28	61.46	71.42	11.16	21.85	31.97	41.36	51.28	61.46	71.42	11.29	21.96	31.87	41.30	51.22	61.20	70.98

(a)

		GMP-G							GMP-NG							GAP-G							GAP-NG						
		Feature Relevance Threshold																											
		10	20	30	40	50	60	70	10	20	30	40	50	60	70	10	20	30	40	50	60	70	10	20	30	40	50	60	70
**Window Length**	**0**	15.36	26.82	38.41	48.46	57.40	65.50	75.14	15.36	26.82	38.41	48.46	57.40	65.50	75.14	15.36	26.82	38.41	48.46	57.40	65.50	75.14	15.84	28.42	40.53	50.31	58.23	65.84	74.84
	**1**	13.41	23.88	33.96	43.75	53.54	63.21	73.80	13.41	23.88	33.96	43.75	53.54	63.21	73.80	13.41	23.88	33.96	43.75	53.54	63.21	73.80	13.08	23.97	34.18	43.77	52.49	62.52	72.04
	**2**	13.18	23.23	33.38	43.21	52.99	63.04	73.52	13.18	23.23	33.38	43.21	52.99	63.04	73.52	13.18	23.23	33.38	43.21	52.99	63.04	73.52	12.95	23.57	33.67	43.61	52.51	62.69	72.22
	**3**	12.64	23.14	33.85	43.51	52.92	62.51	72.41	12.64	23.14	33.85	43.51	52.92	62.51	72.41	12.64	23.14	33.85	43.51	52.92	62.51	72.41	12.61	23.60	33.83	43.89	52.81	62.70	71.67
	**4**	12.11	23.00	33.51	43.05	52.68	62.25	72.06	12.11	23.00	33.51	43.05	52.68	62.25	72.06	12.11	23.00	33.51	43.05	52.68	62.25	72.06	12.31	23.55	33.70	43.51	52.81	62.53	71.63
	**5**	11.86	22.98	33.45	43.25	52.79	62.89	72.74	11.86	22.98	33.45	43.25	52.79	62.89	72.74	11.86	22.98	33.45	43.25	52.79	62.89	72.74	11.90	23.29	33.51	43.53	52.83	62.81	72.13

(b)

		GMP-G							GMP-NG							GAP-G							GAP-NG						
		Feature Relevance Threshold																											
		10	20	30	40	50	60	70	10	20	30	40	50	60	70	10	20	30	40	50	60	70	10	20	30	40	50	60	70
**Window Length**	**0**	17.14	31.05	42.34	52.62	62.10	69.56	77.42	17.14	31.05	42.34	52.62	62.10	69.56	77.42	17.14	31.05	42.34	52.62	62.10	69.56	77.42	17.57	33.11	44.59	55.18	63.06	69.82	77.70
	**1**	14.10	26.05	35.95	45.28	54.81	64.33	74.04	14.10	26.05	35.95	45.28	54.81	64.33	74.04	14.10	26.05	35.95	45.28	54.81	64.33	74.04	13.98	26.40	36.34	46.27	54.45	64.08	72.77
	**2**	13.97	24.35	34.38	43.87	53.54	63.68	73.82	13.97	24.35	34.38	43.87	53.54	63.68	73.82	13.97	24.35	34.38	43.87	53.54	63.68	73.82	13.59	24.72	34.63	45.18	53.40	63.43	72.43
	**3**	13.89	24.95	36.00	45.48	54.91	64.44	74.09	13.89	24.95	36.00	45.48	54.91	64.44	74.09	13.89	24.95	36.00	45.48	54.91	64.44	74.09	13.98	25.74	36.22	46.59	54.80	64.65	73.34
	**4**	13.13	24.29	34.75	43.97	53.78	63.27	73.22	13.13	24.29	34.75	43.97	53.78	63.27	73.22	13.13	24.29	34.75	43.97	53.78	63.27	73.22	13.41	25.11	35.21	45.05	53.85	63.72	72.52
	**5**	13.27	24.90	35.19	44.78	54.34	64.23	74.17	13.27	24.90	35.19	44.78	54.34	64.23	74.17	13.27	24.90	35.19	44.78	54.34	64.23	74.17	13.31	25.34	35.38	45.52	54.35	64.40	73.29

(c)

Table S17: PSSM Motifs - $L_{Grad-RAM}$ Feature Relevance for the sc-PDB pairs across the different feature significance thresholds, window lengths and PSSM Thresholds: a) PSSM Threshold 8; b) PSSM Threshold 9; c) PSSM Threshold 10.

		GMP-G							GMP-NG							GAP-G							GAP-NG						
		Feature Relevance Threshold																											
		10	20	30	40	50	60	70	10	20	30	40	50	60	70	10	20	30	40	50	60	70	10	20	30	40	50	60	70
Window Length	0	8.43	18.00	28.45	38.16	47.81	58.08	68.41	8.43	18.00	28.45	38.16	47.81	58.08	68.41	8.43	18.00	28.45	38.16	47.81	58.08	68.41	8.27	17.97	28.11	38.02	48.02	57.81	67.95
	1	9.37	18.93	29.36	39.14	49.35	59.34	69.40	9.37	18.93	29.36	39.14	49.35	59.34	69.40	9.37	18.93	29.36	39.14	49.35	59.34	69.40	9.21	18.86	29.14	38.92	49.53	59.14	69.22
	2	9.06	18.60	28.75	38.61	48.74	58.73	68.85	9.06	18.60	28.75	38.61	48.74	58.73	68.85	9.06	18.60	28.75	38.61	48.74	58.73	68.85	8.94	18.52	28.75	38.40	48.75	58.65	68.77
	3	9.02	18.74	28.75	38.75	49.07	59.02	69.17	9.02	18.74	28.75	38.75	49.07	59.02	69.17	9.02	18.74	28.75	38.75	49.07	59.02	69.17	8.96	18.67	28.73	38.51	48.99	58.90	69.05
	4	8.93	18.68	28.63	38.56	49.00	59.01	69.10	8.93	18.68	28.63	38.56	49.00	59.01	69.10	8.93	18.68	28.63	38.56	49.00	59.01	69.10	8.84	18.63	28.57	38.28	48.85	58.87	68.96
	5	8.82	18.52	28.39	38.30	48.74	58.86	69.08	8.82	18.52	28.39	38.30	48.74	58.86	69.08	8.82	18.52	28.39	38.30	48.74	58.86	69.08	8.72	18.53	28.34	38.11	48.63	58.74	68.98

(a)

		GMP-G							GMP-NG							GAP-G							GAP-NG						
		Feature Relevance Threshold																											
		10	20	30	40	50	60	70	10	20	30	40	50	60	70	10	20	30	40	50	60	70	10	20	30	40	50	60	70
Window Length	0	8.35	17.93	27.98	37.52	47.79	58.00	68.37	8.35	17.93	27.98	37.52	47.79	58.00	68.37	8.35	17.93	27.98	37.52	47.79	58.00	68.37	8.29	18.05	27.89	37.79	48.32	58.29	67.98
	1	9.95	19.65	29.93	39.47	50.00	59.93	69.77	9.95	19.65	29.93	39.47	50.00	59.93	69.77	9.95	19.65	29.93	39.47	50.00	59.93	69.77	9.71	19.66	29.89	39.35	50.22	59.95	69.69
	2	9.63	19.38	29.48	39.20	49.31	59.57	69.47	9.63	19.38	29.48	39.20	49.31	59.57	69.47	9.63	19.38	29.48	39.20	49.31	59.57	69.47	9.47	19.46	29.68	39.07	49.50	59.58	69.57
	3	9.50	19.46	29.30	39.06	49.23	59.40	69.55	9.50	19.46	29.30	39.06	49.23	59.40	69.55	9.50	19.46	29.30	39.06	49.23	59.40	69.55	9.37	19.42	29.22	38.82	49.09	59.24	69.33
	4	9.31	19.21	29.21	38.94	49.10	59.20	69.17	9.31	19.21	29.21	38.94	49.10	59.20	69.17	9.31	19.21	29.21	38.94	49.10	59.20	69.17	9.21	19.21	29.08	38.59	48.87	58.94	68.96
	5	9.12	19.09	29.13	38.88	49.02	59.15	69.22	9.12	19.09	29.13	38.88	49.02	59.15	69.22	9.12	19.09	29.13	38.88	49.02	59.15	69.22	9.06	19.06	28.94	38.46	48.75	58.81	68.93

(b)

		GMP-G							GMP-NG							GAP-G							GAP-NG						
		Feature Relevance Threshold																											
		10	20	30	40	50	60	70	10	20	30	40	50	60	70	10	20	30	40	50	60	70	10	20	30	40	50	60	70
Window Length	0	8.91	17.71	26.29	34.82	45.68	56.38	67.63	8.91	17.71	26.29	34.82	45.68	56.38	67.63	8.91	17.71	26.29	34.82	45.68	56.38	67.63	8.95	18.09	26.62	35.51	46.22	56.68	67.21
	1	10.38	19.89	29.31	37.88	48.15	58.74	69.06	10.38	19.89	29.31	37.88	48.15	58.74	69.06	10.38	19.89	29.31	37.88	48.15	58.74	69.06	10.04	19.89	29.16	37.65	47.98	58.30	68.68
	2	10.18	20.38	30.44	39.38	49.52	59.87	70.16	10.18	20.38	30.44	39.38	49.52	59.87	70.16	10.18	20.38	30.44	39.38	49.52	59.87	70.16	10.00	20.45	30.48	39.33	49.48	59.64	69.99
	3	10.02	19.97	29.64	38.87	49.13	59.53	69.93	10.02	19.97	29.64	38.87	49.13	59.53	69.93	10.02	19.97	29.64	38.87	49.13	59.53	69.93	9.83	20.09	29.55	38.83	49.05	59.30	69.54
	4	9.62	19.57	29.50	38.83	49.35	59.69	69.92	9.62	19.57	29.50	38.83	49.35	59.69	69.92	9.62	19.57	29.50	38.83	49.35	59.69	69.92	9.50	19.73	29.34	38.70	49.09	59.29	69.55
	5	9.26	18.91	28.73	38.05	48.53	59.07	69.26	9.26	18.91	28.73	38.05	48.53	59.07	69.26	9.26	18.91	28.73	38.05	48.53	59.07	69.26	9.19	19.02	28.52	37.82	48.33	58.72	68.90

(c)

Table S18: PSSM Motifs - $L_{Grad-RAM}$ Feature Relevance for the sc-PDB pairs (with the motifs inside the entire binding region filtered out) across the different feature significance thresholds, window lengths and PSSM Thresholds: a) PSSM Threshold 5; b) PSSM Threshold 6; c) PSSM Threshold 7.

		GMP-G							GMP-NG							GAP-G							GAP-NG						
		Feature Relevance Threshold																											
		10	20	30	40	50	60	70	10	20	30	40	50	60	70	10	20	30	40	50	60	70	10	20	30	40	50	60	70
Window Length	0	11.61	22.69	33.37	41.14	51.09	61.04	71.61	11.61	22.69	33.37	41.14	51.09	61.04	71.61	11.61	22.69	33.37	41.14	51.09	61.04	71.61	11.57	23.25	34.00	42.29	51.05	61.57	70.79
	1	12.08	22.42	32.32	40.24	50.86	61.44	71.81	12.08	22.42	32.32	40.24	50.86	61.44	71.81	12.08	22.42	32.32	40.24	50.86	61.44	71.81	11.80	22.43	32.04	40.09	50.06	60.57	70.69
	2	11.04	21.34	31.16	39.65	49.94	60.68	71.35	11.04	21.34	31.16	39.65	49.94	60.68	71.35	11.04	21.34	31.16	39.65	49.94	60.68	71.35	10.66	21.33	30.89	39.44	49.36	60.02	70.43
	3	10.75	20.80	30.58	39.38	49.64	60.27	70.84	10.75	20.80	30.58	39.38	49.64	60.27	70.84	10.75	20.80	30.58	39.38	49.64	60.27	70.84	10.53	20.97	30.18	39.17	49.20	59.75	69.92
	4	10.07	20.51	30.55	39.55	49.96	60.38	70.76	10.07	20.51	30.55	39.55	49.96	60.38	70.76	10.07	20.51	30.55	39.55	49.96	60.38	70.76	10.02	20.75	30.20	39.37	49.52	59.89	70.14
	5	9.78	19.92	29.87	39.06	49.57	60.40	70.75	9.78	19.92	29.87	39.06	49.57	60.40	70.75	9.78	19.92	29.87	39.06	49.57	60.40	70.75	9.81	20.05	29.54	38.77	49.23	59.87	70.11

(a)

		GMP-G							GMP-NG							GAP-G							GAP-NG						
		Feature Relevance Threshold																											
		10	20	30	40	50	60	70	10	20	30	40	50	60	70	10	20	30	40	50	60	70	10	20	30	40	50	60	70
Window Length	0	12.42	23.55	34.48	43.90	53.96	61.88	72.59	12.42	23.55	34.48	43.90	53.96	61.88	72.59	12.42	23.55	34.48	43.90	53.96	61.88	72.59	12.65	25.55	36.74	45.99	55.23	63.50	72.51
	1	11.95	21.72	31.48	40.07	50.25	60.27	71.72	11.95	21.72	31.48	40.07	50.25	60.27	71.72	11.95	21.72	31.48	40.07	50.25	60.27	71.72	12.00	22.77	32.15	40.58	49.30	59.79	69.82
	2	12.66	22.53	31.93	40.86	50.68	61.50	72.58	12.66	22.53	31.93	40.86	50.68	61.50	72.58	12.66	22.53	31.93	40.86	50.68	61.50	72.58	12.57	23.41	32.45	41.37	50.06	61.01	71.21
	3	11.39	21.27	31.30	40.46	50.41	60.90	71.64	11.39	21.27	31.30	40.46	50.41	60.90	71.64	11.39	21.27	31.30	40.46	50.41	60.90	71.64	11.58	22.20	31.45	41.08	50.21	60.83	70.87
	4	10.62	21.17	31.28	40.41	50.64	61.04	71.45	10.62	21.17	31.28	40.41	50.64	61.04	71.45	10.62	21.17	31.28	40.41	50.64	61.04	71.45	10.97	22.07	31.54	41.09	50.66	61.07	70.97
	5	10.20	20.89	30.88	40.22	50.31	61.21	71.56	10.20	20.89	30.88	40.22	50.31	61.21	71.56	10.20	20.89	30.88	40.22	50.31	61.21	71.56	10.41	21.51	31.04	40.64	50.28	60.93	70.86

(b)

		GMP-G							GMP-NG							GAP-G							GAP-NG						
		Feature Relevance Threshold																											
		10	20	30	40	50	60	70	10	20	30	40	50	60	70	10	20	30	40	50	60	70	10	20	30	40	50	60	70
Window Length	0	13.82	27.06	38.24	47.94	59.12	65.59	74.41	13.82	27.06	38.24	47.94	59.12	65.59	74.41	13.82	27.06	38.24	47.94	59.12	65.59	74.41	14.19	29.39	40.88	51.01	60.47	67.23	74.32
	1	12.36	22.33	32.02	40.03	49.72	59.69	70.22	12.36	22.33	32.02	40.03	49.72	59.69	70.22	12.36	22.33	32.02	40.03	49.72	59.69	70.22	12.93	24.13	33.44	42.11	50.00	60.41	68.93
	2	12.52	21.49	31.13	39.85	49.75	60.74	71.74	12.52	21.49	31.13	39.85	49.75	60.74	71.74	12.52	21.49	31.13	39.85	49.75	60.74	71.74	12.26	22.56	31.94	41.32	49.30	60.54	70.10
	3	12.25	22.08	32.74	41.74	51.75	62.03	72.81	12.25	22.08	32.74	41.74	51.75	62.03	72.81	12.25	22.08	32.74	41.74	51.75	62.03	72.81	12.46	23.30	33.10	43.11	51.29	62.14	71.87
	4	11.41	21.44	31.47	40.23	50.46	60.70	71.60	11.41	21.44	31.47	40.23	50.46	60.70	71.60	11.41	21.44	31.47	40.23	50.46	60.70	71.60	11.72	22.48	31.89	41.42	50.17	60.87	70.63
	5	11.15	21.68	31.47	40.59	50.64	61.17	71.79	11.15	21.68	31.47	40.59	50.64	61.17	71.79	11.15	21.68	31.47	40.59	50.64	61.17	71.79	11.31	22.33	31.57	41.39	50.29	61.02	70.60

(c)

Table S19: PSSM Motifs - $L_{Grad-RAM}$ Feature Relevance for the sc-PDB pairs (with the motifs inside the entire binding region filtered out) across the different feature significance thresholds, window lengths and PSSM Thresholds: a) PSSM Threshold 8; b) PSSM Threshold 9; c) PSSM Threshold 10.

References

[1] Tapio Pahikkala, Antti Airola, Sami Pietilä, Sushil Shakyawar, Agnieszka Szwajda, Jing Tang, and Tero Aittokallio. Toward more realistic drug-target interaction predictions. *Briefings in Bioinformatics*, 16(2):325–337, 04 2014.

[2] Jooyong Shim, Zhen-Yu Hong, Insuk Sohn, and Changha Hwang. Prediction of drug-target binding affinity using similarity-based convolutional neural network. *Scientific Reports*, 11(1):4416, Feb 2021.

[3] Tong He, Marten Heidemeyer, Fuqiang Ban, Artem Cherkasov, and Martin Ester. Simboost: a read-across approach for predicting drug–target binding affinities using gradient boosting machines. *Journal of Cheminformatics*, 9(1):24, Apr 2017.

[4] Hakime Öztürk, Arzucan Özgür, and Elif Ozkirimli. DeepDTA: deep drug-target binding affinity prediction. *Bioinformatics*, 34(17):i821–i829, 09 2018.

[5] Thin Nguyen, Hang Le, Thomas P Quinn, Tri Nguyen, Thuc Duy Le, and Svetha Venkatesh. GraphDTA: Predicting drug-target binding affinity with graph neural networks. *Bioinformatics*, 10 2020. btaa921.

[6] Karim Abbasi, Parvin Razzaghi, Antti Poso, Massoud Amanlou, Jahan B Ghasemi, and Ali Masoudi-Nejad. DeepCDA: deep cross-domain compound-protein affinity prediction through LSTM and convolutional neural networks. *Bioinformatics*, 36(17):4633–4642, 05 2020.

[7] Helen M. Berman, John Westbrook, Zukang Feng, Gary Gilliland, T. N. Bhat, Helge Weissig, Ilya N. Shindyalov, and Philip E. Bourne. The Protein Data Bank. *Nucleic Acids Research*, 28(1):235–242, 01 2000.

[8] Dassault Systèmes. BIOVIA Discovery Studio, 2021.

[9] Noel M. O'Boyle, Michael Banck, Craig A. James, Chris Morley, Tim Vandermeersch, and Geoffrey R. Hutchison. Open Babel: An open chemical toolbox. *Journal of Cheminformatics*, 3(1):33, Oct 2011.

[10] Jerome Eberhardt, Diogo Santos-Martins, Andreas F. Tillack, and Stefano Forli. AutoDock Vina 1.2.0: New Docking Methods, Expanded Force Field, and Python Bindings. *Journal of Chemical Information and Modeling*, 61(8):3891–3898, Aug 2021.

[11] Andrea Volkamer, Daniel Kuhn, Thomas Grombacher, Friedrich Rippmann, and Matthias Rarey. Combining global and local measures for structure-based druggability predictions. *Journal of Chemical Information and Modeling*, 52(2):360–372, Feb 2012.

[12] Schrödinger, LLC. The PyMOL Molecular Graphics System, Version 2.5. May 2021.

[13] The UniProt Consortium. UniProt: the universal protein knowledgebase in 2021. *Nucleic Acids Research*, 49(D1):D480–D489, 11 2020.

[14] Stephen F. Altschul, Warren Gish, Webb Miller, Eugene W. Myers, and David J. Lipman. Basic local alignment search tool. *Journal of Molecular Biology*, 215(3):403–410, 1990.

[15] Simone Lemeer, Andrej Bluwstein, Zhixiang Wu, Julia Leberfinger, Konrad Müller, Karl Kramer, and Bernhard Kuster. Phosphotyrosine mediated protein interactions of the discoidin domain receptor 1. *Journal of Proteomics*, 75(12):3465–3477, Jun 2012.
